# Interactions between faces and visual context in emotion perception: A meta-analysis

**DOI:** 10.3758/s13423-025-02678-6

**Published:** 2025-04-03

**Authors:** Ben A. Steward, Paige Mewton, Romina Palermo, Amy Dawel

**Affiliations:** 1 School of Medicine and Psychology, The Australian National University, Canberra, ACT 2601 Australia; 2 School of Psychological Science, The University of Western Australia, Perth, WA 6009 Australia

**Keywords:** Affective integration, Facial primacy, Nonverbal communication

## Abstract

Long-standing theories in emotion perception, such as basic emotion theory, argue that we primarily perceive others’ emotions through facial expressions. However, compelling evidence shows that other visual contexts, such as body posture or scenes, significantly influence the emotions perceived from faces and vice versa. We used meta-analysis to synthesise and quantify these effects for the first time, testing if faces have primacy over context after accounting for key moderators. Namely, the emotional congruency and clarity of the stimuli. A total of 1,020 effect sizes from 37 articles and 3,198 participants were meta-analysed using three-level mixed-effects models with robust variance estimation. Both visual context and faces were found to have large effects on emotion labelling for the other (*g*_*av*_ > 1.23). Effects were larger when visual context and faces signalled different (incongruent) rather than the same (congruent) emotions and congruent effects were moderated by how clearly stimuli signalled the target emotion. When these factors were accounted for, faces were no more influential in altering emotion labelling than body postures or body postures with scenes. The findings of this review clearly evidence the integrative nature of emotion perception. Importantly, however, they also highlight that the influence of different emotion signals depends on how clearly they signal an emotion. Future research needs to account for emotional congruency and signal clarity.

**Supplementary Information:**

The online version contains supplementary material available at 10.3758/s13423-025-02678-6.

## Introduction

We often use other people’s facial expressions as a reference point to learn about and understand events in our physical and social worlds. For example, a parent’s negative facial expression in response to a spider may help a child learn to avoid touching them (Sorce et al., 1985). However, facial expressions are not all there is to see. The child may also use other information, including visual cues such as their parent’s body posture or the spider itself, to interpret their parent’s facial expression and judge the overall emotional tone of the situation. While several theoretical traditions emphasise the primacy of facial expressions in emotion perception (e.g., Ekman, 1989; Ekman & Friesen, 1976), there is now compelling evidence that visual *context*, such as body posture and visual scenery, influences the perception of emotion in facial expressions (Aviezer et al., 2008, 2017; Aviezer, Hassin, & Bentin, 2012; Aviezer, Trope, & Todorov, 2012a, 2012b; Barrett et al., 2011; Civile & Obhi, 2016; Hassin et al., 2013; Israelashvili et al., 2019; Ko et al., 2011; Kret et al., 2013; Lecker et al., 2020; Masuda et al., 2008; Nelson & Mondloch, 2017; Namba et al., 2020; Righart & de Gelder, 2008a; Xiao et al., 2016). In addition to the influence of visual context on the perception of emotion in faces (termed the *effect of context*), there is also an *effect of faces* whereby facial expressions influence the perception of emotion in the surrounding visual context (Hümmer et al., 2021; Kret et al., 2013; Lecker et al., 2020; Reschke & Walle, 2021). The *effect of faces* is consistent with alternative theories of constructed emotion that propose emotion perception is established from integrating available information (Barrett et al., 2019).

While there is now broad agreement that both faces and visual context mutually influence emotion perception in one another (Reschke & Walle, 2021), it is unknown how much they do so. This mutual influence may also be moderated by stimulus-level factors that have not been previously considered, such as signal clarity and emotional congruence. The present study aims to provide the first meta-analytic synthesis of individual studies testing these effects and to investigate key moderators.

A critical question is whether faces or visual context are more influential in altering emotion labelling. Although individual studies have started to address this question (Chen & Whitney, 2019; Lecker et al., 2020), they have not controlled for important confounds, such as how clearly a stimulus signals the intended emotion. One possibility borne out of the theory of constructed emotion is that whichever stimulus is most clear will be the most influential, irrespective of whether that stimulus is a face or surrounding visual context. Alternatively, people may give facial information more weight than other types of information, in line with basic emotion theory (Ekman, 1989; Ekman & Friesen, 1976; Tracy & Randles, 2011). Another important question is how the effects of faces and context vary when emotional information is congruent (e.g., a fearful face with a cowering body) compared to incongruent (e.g., a happy face with a cowering body). Identifying if and how faces and context differentially influence each other when resolving differences in congruency provides valuable insight into how people navigate the social world and the uncertainty of others’ emotional displays (Abramson et al., 2017).

## Relevant theoretical background

Longstanding theoretical traditions emphasise the primacy of facial expressions in emotion perception via claims that certain basic expressions are universal, such as happiness, sadness, and fear (Ekman, 1989; Ekman & Friesen, 1976). Basic emotions theory, for instance, proposes that a small set of basic emotions are displayed in consistent ways, which are diagnostic of a person’s emotional state (Ekman, 1989; Ekman et al., 1972; Tracy & Randles, 2011). This idea suggests that the information contained in a face can be directly ‘read’ as a reliable signal of emotion, irrespective of the surrounding context. For example, if a parent looks happily at brussels sprouts, their child may infer they like it. However, although evidence continues to show that faces per se strongly influence emotion perception (Calvo & Nummenmaa, 2016), traditional basic emotions theory cannot account for the influence of context on facial emotion perception. Although more recent advances in basic emotions theory do account for the influence of context on facial emotion perception (e.g., Keltner et al., 2019), prototypical facial expressions are still conceptualised as signalling specific emotions.

A contemporary alternative that accounts for the effect of context is the theory of constructed emotion (Barrett, 2017; Barrett et al., 2019). This theory proposes that people make a ‘best guess’ about others’ emotions using all available information, integrating facial expressions, body posture, and any other context. Thus, rather than ‘reading’ faces directly, people also draw on the information available alongside facial cues to interpret someone’s emotional state. For instance, in the example of the parent looking happily at brussels sprouts, the integration of body posture signalling disgust may suggest the parent does not actually like brussels sprouts (but is pretending to do so in front of their child). This framework suggests both faces and context influence emotion perception, as supported by recent empirical evidence (Hümmer et al., 2021; Lecker et al., 2020; Reschke & Walle, 2021). Although the theory of constructed emotion does not make any explicit suggestion about which type of information should be most influential, it does suggest differences in expressive displays (e.g., differences in facial expressions across emotional episodes) can be informative (Gendron & Barrett, 2017). Therefore, emotion perception may be influenced by the clarity with which faces, or visual context signal their intended emotion.

## Potential influence of emotional congruence

The emotional information present in faces and visual context can be *congruent*, showing the same emotion, or *incongruent*, showing different emotions. For example, at a birthday party most children will be happy and smiling (congruent emotional display), but some may express anxiety in social settings, which would be incongruent with the broader visual context of the fun-looking party. Existing research demonstrates that when face and visual context information are congruent people are more likely, and faster, to choose the intended emotion label for the face or context compared to when they are incongruent (Bjornsdottir et al., 2017; Civile & Obhi, 2016; Fenske & Eastwood, 2003; Foul et al., 2018, 2022; Lecker & Aviezer, 2021; Noh & Isaacowitz, 2013). Additionally, compared to when stimuli are shown in isolation, people are more likely to choose the intended emotion label when face-context pairings are emotionally congruent (Aviezer et al., 2008; Lecker et al., 2020; Reschke & Walle, 2021; Righart & de Gelder, 2008a, 2008b; Willis et al., 2011). Conversely, the same set of studies show accuracy is lower when face-context pairings are incongruent. For example, Righart and de Gelder (2008b) found observers were more likely to label a facial expression as its intended emotion (e.g., disgust) when it was presented with scenes conveying congruent emotions (e.g., revolting rubbish). However, presenting that same face with scenes conveying incongruent emotions (e.g., pleasant flowers) had the inverse effect, decreasing how often observers labelled the face as its intended emotion. Interestingly, these effects were found despite observers being instructed to ignore the background scene, highlighting the automaticity with which this integration happens. Moreover, when the facial expression was miscategorised, observers often chose an emotion that matched the background scene. Similar effects have also been found for bodies. In their seminal work, Aviezer et al. (2008) found pairing facial expressions conveying one emotion when seen in isolation (e.g., disgust) with body postures conveying incongruent emotions (e.g., anger, sadness) caused observers to miscategorise the face as showing the emotion conveyed by the body, even though observers were told to focus on the face. Overall, these findings suggest congruent information enhances the target emotion signal, whereas incongruent information can lead to categorical shifts in emotion perception.

While evidence for the influence of emotional congruence is robust, an important question remains: how much does congruent and incongruent information influence the size of the effects of faces and context? There is reason to predict incongruent information may be more influential than congruent information. Statistically, labelling agreement is closer to ceiling than floor for many popular isolated face and context stimuli, which leaves less room for congruent pairings to increase agreement than for incongruent pairings to decrease agreement. A second, more substantive, reason is that humans are particularly sensitive to information that errs from what is predicted, as occurs in incongruent stimulus pairings (Davis et al., 2020; Hamm et al., 2002; Sambrook & Goslin, 2015; Truman & Mudrik, 2018). This increased sensitivity would suggest a greater weighting of unexpected information from incongruent stimuli during perception, leading to larger shifts in perceptual judgment.

## Potential influence of signal clarity

The clarity of stimuli to signal their intended emotion is rarely considered in the literature, despite being central to real-life perception. Evidence indicates signal clarity has an important role in emotion perception. In one key study, Aviezer, Trope, and Todorov (2012a) found observers could not identify the valence of real-life, isolated facial expressions that were captured in intense positive or negative situations, such as a professional tennis player winning or losing a point. However, pairing these unclear faces with winning or losing body postures caused observers to rate the faces as positive or negative, respectively. This example illustrates how additional clear signals may help resolve ambiguous information in emotion perception.

Less clear visual cues may have less influence on perceptual judgments because the stimulus creates a weaker activation of the semantic network associated with the target label (weak within-cluster connectivity), while also activating networks containing other target labels (stronger between-cluster connectivity). Semantic network theory suggests information is represented by nodes that are interconnected within clusters of related information. For example, an anger cluster would include nodes containing information about angry faces, contexts, and words. If the visual cues do not clearly signal their emotion (e.g., anger), other clusters (e.g., disgust) may be activated more easily (Bartel et al., 2020; Berthet et al., 2011; Marko & Riečanský, 2021) and cause perceptual signals from unclear stimuli to be associated with more than one emotion label (Trope, 1986). Supporting this prediction, Van den Stock et al. (2007) found the effect of body posture on emotion judgments for faces increased as facial expressions became less clear. Moreover, Karaaslan et al. (2020) found when stimuli were presented briefly (33 ms), visual context influenced emotion judgments only for low clarity facial expressions.

Although signal clarity has received limited investigation in the past, it is important to consider how much it may influence our emotion judgements. There is reason to expect as the clarity-difference between the target and the added stimulus increases we would see a larger influence from the added stimulus. A potential reason for this is that by having a clearer signal, the added stimulus would have greater activation of the semantic network of its intended emotion. This greater semantic network activation would then lead to larger shifts in our perceptual judgements.

## The present study

The present study aimed to characterise the size of the effects of context and faces on emotion perception and to investigate factors that moderate them. Our analyses focus on synthesising evidence from visual context specifically (e.g., bodies, visual scenes) as sufficient individual studies exist to warrant this integration. We did not include social vignettes to focus on sensory-level, bottom-up processing and integration of affective stimuli.

Of central importance to this review was establishing whether the *effect of context* or the *effect of faces* was larger once moderators (i.e., the emotional congruence of paired stimuli and their relative clarity) were accounted for. While previous studies have investigated the effect of both faces and context, it is unknown which, if either, is larger (e.g., Chen & Whitney, 2020; Lecker et al., 2020; Reschke & Walle, 2021). Critically, these studies have not comprehensively considered the role of emotional congruency and signal clarity. We also investigated the potential role of stimulus-level factors as moderators, including the emotion category of the stimuli, the type of visual context (bodies, visual scenery), presentation duration, and if stimuli were dynamic or static. Emotion category (e.g., anger, disgust, happiness, etc.) was of interest because some emotions may be signalled more distinctly by faces (e.g., fear) and others by visual context (e.g., anger; Lecker et al., 2020)**.** Context type was of interest as bodies and visual scenes each provide unique emotional information (Chen & Whitney, 2020, 2021; Reschke & Walle, 2021). Presentation duration (i.e., viewing time) was of interest as both faces and bodies elicit fast, as well as subconscious, processing of emotional displays (Martinez et al., 2016; Palermo & Rhodes, 2007; Stienen & de Gelder, 2011). Dynamic versus static presentation was of interest because real-life expressions are typically moving, and dynamic stimuli have shown stronger effects (Krumhuber et al., 2013), but most research uses static images (Dawel et al., 2021).

In summary, and in line with our pre-registered review protocol, we aimed to evaluate the size of the effects of context (bodies, scenes) and faces in emotion perception. We then aimed to test if emotionally congruent and incongruent pairings affect emotion perception agreement differently, if relatively clearer stimuli affect emotion perception agreement more, and if changes in emotion perception agreement differ by emotion category, presentation duration, or presentation format (i.e., if stimuli are static/still or moving/dynamically presented).

## Methods

The review was pre-registered on the Open Science Framework (https://osf.io/mn7qk/), and conducted following the PRISMA Statement and Checklist (Page et al., 2021).

### Operationalising emotion perception

This review focuses on emotion labelling, operationalised as how often observers agreed with the emotion label pre-assigned to each stimulus. While these judgements are often termed *accuracy* in the literature, we prefer to use the term *agreement* to reflect consensus (i.e., agreement) between observers’ perceptual judgement and labels determined by other perceptual criteria (e.g., expert ratings), rather than accuracy in judging the expressors’ emotional experience (see Barrett et al., 2019). In addition to emotion labelling agreement, the review included emotion ratings and speed of labelling as measures of emotion perception. However, we found the vast majority of effect sizes were for labelling agreement. Additionally, there were often insufficient rating and reaction time effects for moderator analyses. Thus, we focus on labelling agreement in the main text and present complementary meta-analyses for ratings and reaction times in Online Supplement Materials 8 and 9.

### Operationalising signal clarity

In the present study, we operationalised signal clarity as the agreement in observers’ emotion judgements when stimuli were presented in isolation (i.e., the percentage of observers who ‘correctly’ labelled a face or visual context shown in isolation, without the other). Previous research has shown that the signal clarity of facial expressions is positively associated with inter-observer emotion-labelling agreement (Matsumoto et al., 2009). As such, labelling agreement can provide a group-level measure of signal clarity. For brevity, we use the term ‘clarity’ in place of ‘signal clarity’ henceforth. As we were specifically interested in the effect of adding one stimulus to another, we calculated the difference in clarity scores (for isolated stimuli) between the target and the added stimulus (i.e., observer agreement for isolated added stimuli minus observer agreement for isolated targets).

### Study eligibility criteria

Articles were eligible for inclusion if they used a behavioural measure of emotion perception (labelling agreement, ratings such as how happy or how positive/negative, and/or reaction times) for face or visual context stimuli when they were presented in isolation *and* when they were presented together at the same time (i.e., simultaneous presentation). Articles that tested non-visual contexts only (e.g., written vignettes) were excluded. We required study participants to be at least 4 years old, consistent with the development of basic facial emotion discrimination (Widen, 2013). We excluded data from clinical populations to provide a more precise effect estimate for the general population and reduce heterogeneity to better assess the moderators. Articles had to be published in peer-reviewed journals, with the full-text available in English. Articles that presented only qualitative data were excluded. Searches included all available publication years.

### Search strategy

We searched the titles, abstracts, and keywords of three databases (PsycInfo, Scopus, Web of Science) to identify articles that included emotion perception (‘emotion*’ or ‘valenc*’ within three words of ‘perception’, ‘identif*’, ‘label*’, ‘recogni*’, ‘judg*’, ‘content’; or ‘valenc*’ within three words of ‘positiv*’, ‘negativ*’), faces (‘face’; ‘faces’; ‘facial’; ‘expression*’) and visual context (‘context*’; ‘body language’; ‘posture’; ‘pose’; ‘gesture’; ‘scene’; ‘background’; see Online Supplementary Material 1 for full syntax). The databases were accessed at the start of May 2022, following registration of the study protocol on Open Science Framework (OSF). Following initial screening, we also searched for additional eligible titles in the reference lists of included articles and relevant review articles, and the publication lists of authors with three or more eligible articles, using Google Scholar, ORCiD, university webpages, and Web of Science.

### Article screening

Duplicate articles were identified and removed using Endnote’s duplicate function, or manually by reviewers during screening. Titles and abstracts from the database searches were screened independently by BS and either PM or a second reviewer (see *Acknowledgements*). Reference and publication lists were screened by BS. The full text of all retained articles was screened independently by BS and PM. Conflicting decisions were discussed and resolved by the two reviewers at both stages, arbitrated by AD if required.

### Data extraction

BS and PM independently extracted all data. Data included: (1) means and standard deviations (or effect sizes if raw data were not available) for labelling agreement, emotion ratings, and/or reaction times, for stimuli presented in isolation (faces *or* visual context) and together (faces *with* visual context); (2) emotion categories for each face and context condition (used to determine emotional congruency); (3) type of visual context (e.g., bodies, scenes); (4) presentation duration; (5) whether stimuli were dynamic or static; (6) other experimental details (e.g., stimulus databases, number of response options) and (7) participant details (e.g., *N*, sex, age). We extracted numerical data directly from the article or its supplementary materials where possible. If only graphical data were available, we used WebPlotDigitzer to estimate data (Rohatgi, 2021). If a study appeared to collect the required data but did not report it, BS requested the data from the authors (minimum of three attempts). Articles that did not provide sufficient data were excluded from the meta-analysis. The reliability of the data extraction process was verified by comparing the data extracted by BS to that extracted by PM. A total of 61,929 cells of data were extracted, of which only 2.2% differed between the two extractors and were corrected prior to analysis. Cell data that differed was corrected collaboratively by BS and PM who reviewed the cells’ data and re-extracted the correct data together.

### Effect measures

**Fig. 1:**
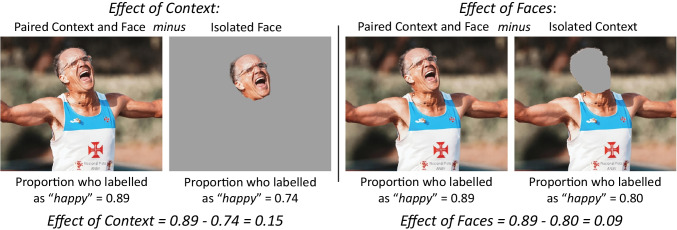
**Calculation of the effect of context and the effect of faces**. Photograph from RUN 4 FFWPU, care of Pexels

The primary effects of interest, the *effect of context* and the *effect of faces,* were calculated as the mean differences between isolated and paired stimuli for faces and visual context, respectively. An example of both the *effect of context* and the *effect of faces* can be seen in Fig. 1. For example, a face and a body can be presented in three ways: the isolated face, the isolated body, and the paired face + body. The *effect of context* is the mean difference in response between the isolated face and paired face + body conditions, measuring how emotion perception changes when a body is added to a face shown in isolation. Alternatively, the *effect of faces* is the mean difference in response between the isolated body and paired face + body conditions, measuring how emotion perception changes when a face is added to a body shown in isolation. The mean difference effect size, Hedge’s *g*_*av*_*,* was calculated using the extracted means and standard deviations and following the recommendations of Lakens (2013; see Online Supplementary Material 3 for calculation).

## Results

### Included articles

**Fig. 2:**
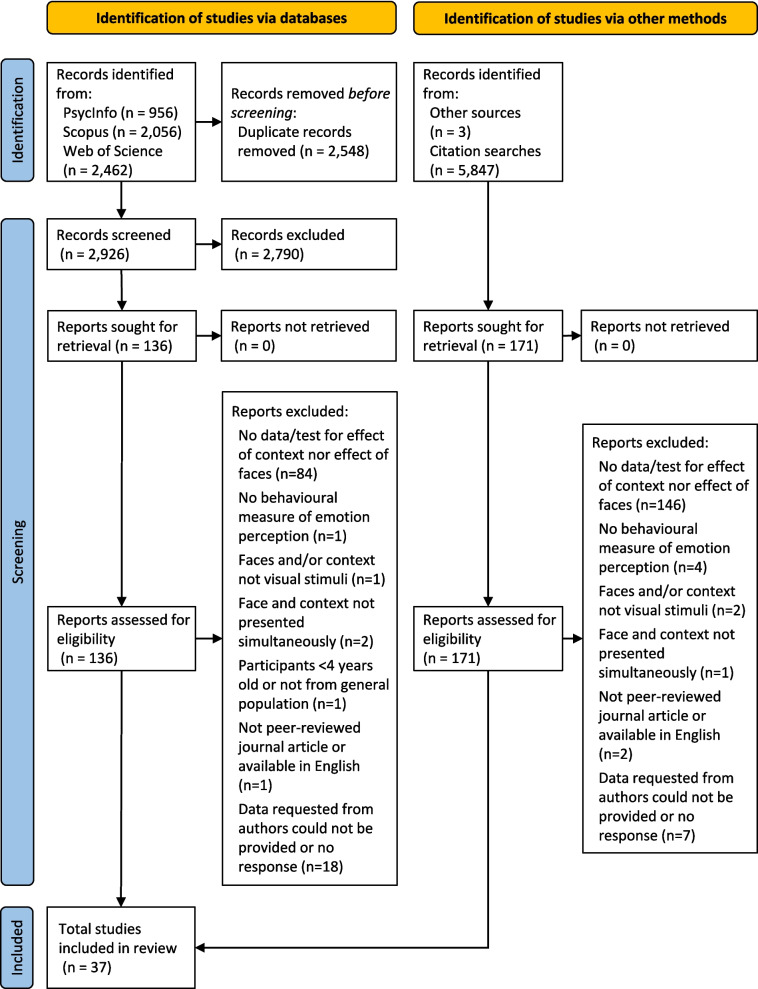
**PRISMA (Preferred Reporting Items for Systematic Reviews and Meta-Analyses) flow diagram**

Sixty-two articles met inclusion criteria, 37 of which had data available for meta-analysis. Figure 2 presents the PRISMA flow diagram showing the number of articles included and excluded at each stage of the search. Inter-rater reliability for title and abstract screening was .51κ, indicating moderate agreement, and .82κ for full-text review, indicating near-perfect agreement (Landis & Koch, 1977). The vast majority of effects were for labelling agreement (n = 752 effects from 30 articles, see Online Supplementary Material 2), with fewer for ratings (n = 123 effects from nine articles, see Online Supplementary Material 8) and reaction time (n = 145 effects from nine articles, see Online Supplementary Material 9). Unfortunately, there were insufficient data to build all intended models for ratings or reaction times, which meant we were unable to explore all moderators for these outcomes. However, a *reduced model* (which assessed only congruency and effect type) was built for both. No significant effects were identified, but there were numerical trends in both models. In the ratings *reduced model*, congruency effects were numerically in the expected direction. However, the effect of context increased observers’ ratings, whilst the effect of faces decreased observers’ ratings. The *reduced model* for reaction times showed numerically slower responses for paired compared to isolated stimuli for all effects. As there was insufficient data to build a testable model with clarity-difference and other moderators, and the *reduced models* were underpowered, we focus on labelling agreement in the main text and present meta-analyses for ratings and reaction times in Online Supplementary Materials 8 and 9.

### Meta-analyses

#### Data screening and quality checks

##### Outliers

 To interrogate the influence of outliers on results, all analyses were run three times (see next section for model analysis plan): (1) all effect sizes included, (2) influential outliers at the effect size level removed, and (3) influential outliers at the study level removed (i.e., where a study was an influential outlier, all effect sizes for that study were removed). Outliers were identified using studentised deleted residuals. Influential effects were identified using Cook’s Distance and DFBETA statistics. Influential outliers were instances where both studentised deleted residuals and either Cook’s Distance or DFBETA statistics were significant (see Online Supplementary Material 6 for figures). Two effect sizes failed to have studentised deleted residuals calculated and so were, conservatively, treated as influential outliers.^1^ No other effect sizes were influential outliers. Two studies were identified as influential outliers. Removing influential outliers did not impact the primary results and, thus, we report models with all articles included to represent the literature as accurately as possible. Models with and without influential outliers are presented in the supplemental r-markdown.

##### Publication bias

To assess for publication bias, we used the precision-effect test and precision effect estimate with standard errors framework (PET-PEESE; Stanley & Doucouliagos, 2014). PET and PEESE are calculated by entering the effect size standard error (PET) or the effect size variance (PEESE) as the only predictor in the model. Potential publication bias is indicated when the PET model intercept is significant. Under this framework the PET model intercept is reported when it is equal to or less than zero; otherwise, PEESE is reported. Reporting in this way maximises the accuracy of estimates (Stanley & Doucouliagos, 2014).

Using the PET-PEESE framework, we detected publication bias in the initial baseline model (PET: *β* = 3.12, *p* = .003; PEESE: *β* = −0.31). The PEESE intercept suggests the effects reported in the literature may be inflated, and so, the meta-analytic effect size estimates should be interpreted with caution.

##### Confidence in the evidence

Overall confidence in the evidence was moderate. Confidence in the evidence was based on four of the eight criteria from the Grading of Recommendations, Assessment, Development, and Evaluations (GRADE) framework (Guyatt et al., 2008). Namely, imprecision, inconsistency, publication bias, and large magnitude of effect (Guyatt, Oxman, Kunz, Brozek, et al., 2011; Guyatt, Oxman, Kunz, Woodcock, et al., 2011; Guyatt, Oxman, Montori, et al., 2011; Guyatt, Oxman, Sultan, et al., 2011). Confidence in the evidence was initially rated as high due to the adequacy of sample sizes (exceeding the optimal information size) and the large magnitude of the effects of primary interest (*g*_*av*_ = 0.70 to 3.23). However, this confidence was diminished by the high levels of heterogeneity among effect sizes (QEs > 16,340), suggesting inconsistency in the underlying effects across included studies, and the indication of potential publication bias (PEESE: *β* = −0.31). The remaining four GRADE criteria, such as dose–response gradient, are specific to clinical research and thus were not relevant to the present study. Justifications for the GRADE criterion decisions are presented in Online Supplementary Material 7.

#### Analysis strategy

Meta-analyses used three-level mixed effects models performed with the metafor (Viechtbauer, 2010) package in R version 4.4.1 (R Core Team, 2023). This approach catered to dependencies between effects (Cheung, 2014; Fernández-Castilla et al., 2021) as all studies reported multiple effects (e.g., one for each category of emotion tested). Random effects were hierarchically nested in the models such that effect sizes were pooled at the participant level and then nested within the article they came from. This multi-level modelling explicitly modelled within-article heterogeneity and data dependencies. To optimise modelling of the dependency structure further, we also applied robust variance estimation (RVE; Fernández-Castilla et al., 2021; Hedges et al., 2010; Pustejovsky, 2022) using the clubSandwich package in R (Pustejovsky, 2022). While the use of RVE was not specified in our pre-registered protocol, this approach better estimates unbiased effects (Hedges et al., 2010; Tipton & Pustejovsky, 2015) and meta-analytic models with and without RVE produced the same direction of results (see supplemental r-markdown). All models were fitted using restricted maximum likelihood estimation to provide approximately unbiased estimates of model heterogeneity (Viechtbauer, 2005).

Baseline models without any predictors assessed if the three-level structure fit the data better than a two-level structure without nested effects. To this end, we built two baseline models (*k* = 30 articles and *n* = 752 effect sizes for both models): the *initial baseline model*, using the original effect sizes, and the *score-adjusted model*. The *score-adjusted model* reverse-scored effect sizes when facial expressions and context were emotionally incongruent (i.e., showing different emotions, with these effects almost always being negative) so their absolute size could be directly compared with effect sizes for emotionally congruent pairings (which were almost always positive).

**Fig. 3:**
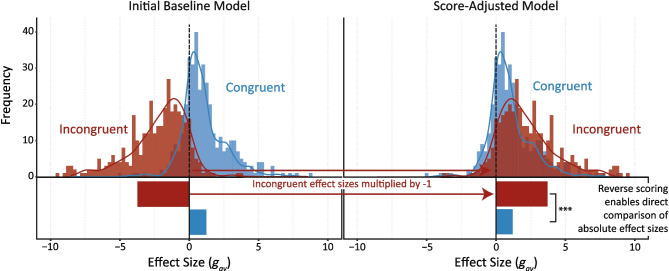
**Illustration of** **reverse** **scoring the** **incongruent** **effects**. The histogram bars represent the frequency of the calculated effect sizes, binned by 0.25*g*_*av*_. Each curve is the smoothed density estimate of the histograms. Effect sizes with Hedges *g*_*av*_ of -/ + 10 are not represented in this figure to optimise the visibility of most of the data

**Fig. 4:**
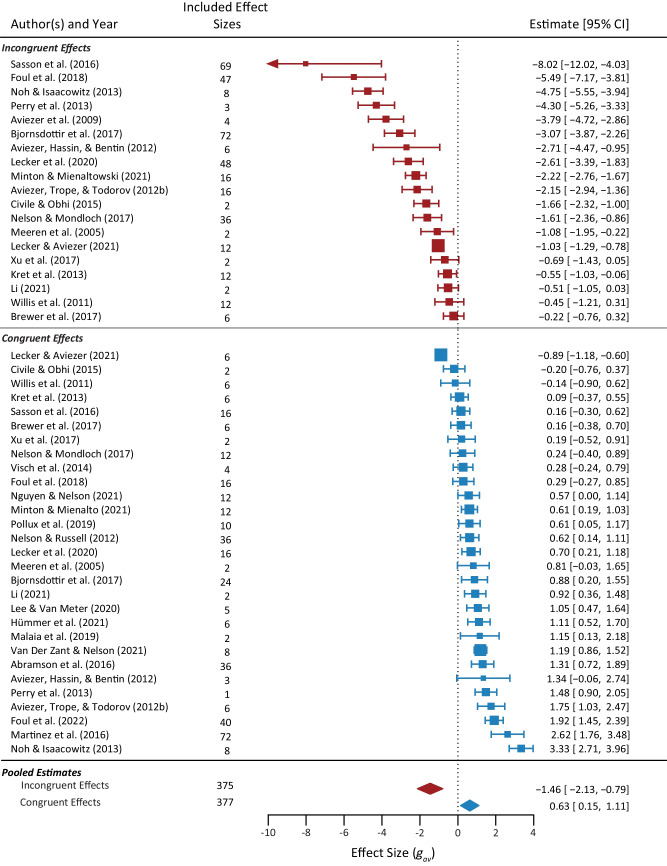
**Forest plot of incongruent (red) and congruent (blue) effect size estimates pooled by study**. Arrows indicate that the 95% confidence interval falls outside of the included scale

Figure 3 illustrates this reverse-scoring of incongruent effects. For both baseline models, the three-level structure fit the data better than the two-level structure (*p*s < .001; see Online Supplementary Material 4), which justified the modelling of dependencies in the data. Figure 4 presents the estimated congruent (in blue) and incongruent (in red) effects per study.

The intercept of the main meta-analytic models estimates effect sizes when all factors are at their reference level, as indicated by the note below each model table. For instance, for the model presented in Table 1, the reference levels for effect type and congruency are the *effect of context* and congruent effects respectively. Importantly, the model coefficients represent each term’s effect while holding all other factors at their reference level. For instance, the congruency effect in Table 1 estimates the effect of incongruent relative to congruent effects with effect type held at its reference level (i.e., for the *effect of context*). Since this approach does not directly estimate factor main effects, we supplemented our primary models with omnibus Wald-type tests to estimate the main effect of each factor (indicated by *F* values). Where interaction effects were significant in the meta-analytic models (indicated by *t* values), we also ran post hoc pairwise Wald-type comparisons to interrogate simple effects. In some circumstances we compared effect sizes to zero; for instance, to test if the *effect of faces* and the *effect of context* were significantly different from zero. For these comparisons, we used an alternative parameterization of the meta-analytic models with the intercept removed.

**Table 1:** **Main model results (k = 24, n = 701)**

	**Vs. reference levels**				**Wald omnibus**	
**Model term**	***g***_***av***_**[95% CI]**	**SE**	***t***	***p***	***F***	***p***
Intercept	0.68 [0.24, 1.13]	0.21	3.21	.005		
Effect of faces (vs. ref = Effect of context)	0.04 [−0.23, 0.31]	0.12	0.31	.762	1.86	.232
Incongruent (vs. ref = Congruent effects)	1.31 [0.52, 2.09]	0.36	3.67	.004	10.38	.012
Clarity-difference (vs. ref = 0)	4.43 [3.55, 5.31]	0.36	12.23	< .001	47.96	.004
Effect Type*Congruency	3.06 [−0.69, 6.81]	1.64	1.87	.097		
Effect Type*Clarity-difference	1.65 [−1.69, 5.00]	1.44	1.15	.285		
Congruency*Clarity-difference	−5.80 [−11.12, −0.49]	2.03	−2.86	.038		

Reference levels for the intercept are effect type = effect of context, congruency = congruent effects, and clarity-difference = 0. Wald omnibus = estimated main effect of factor using supplemental omnibus Wald-type test. Model fit statistics = I2_(2)_/ I2_(3)_ = .17/.81; Q = 16,339; Likelihood Ratio Test: χ^2^(3) = 132.20, *p* < .001.

To test if effect sizes differed by congruency and/or effect type, we built our main meta-analytic models. Our score-adjusted *main model* (*k* = 24, *n* = 701) included the following predictors: effect type (two levels: effect of context, effect of faces),^2^ congruency (two levels: congruent, incongruent), clarity-difference scores, and their interaction terms (i.e., effect type*congruency, effect type*clarity-difference, and congruency*clarity-difference). Clarity-difference scores were calculated as mean labelling agreement for added stimuli (when presented in isolation) minus mean labelling agreement for target stimuli (when presented in isolation). This approach quantified whether the emotional signal of the added stimuli were more or less clear than the signal of the target stimuli. We also re-analysed the data with a *reduced model* that excluded clarity-difference terms to allow for the inclusion of additional studies that did not provide this data (*k* = 30, *n* = 752). However, results from the *reduced model* are consistent with the *main model* and thus we report the *reduced model* results in Online Supplementary Material 5 (Table S5a).

Finally, we tested the role of the remaining moderators by adding them individually to the *main model*. The additional moderators were emotion category (anger/disgust/fear/happy/sad for target stimuli: *k* = 21 and *n* = 589; and for added stimuli: *k* = 21 and *n* = 591), presentation duration (limited vs. unlimited presentations, *k* = 22 and *n* = 557; and limited presentation length in ms, *k* = 10 and *n* = 225), and presentation format (dynamic vs. static, *k* = 24 and *n* = 701). Context type (body vs. visual scene) was not included as a moderator as planned because all ‘scene’ stimuli included bodies**.**

#### Do effect sizes differ for emotionally congruent and incongruent pairings?

The addition of incongruent information decreased labelling agreement, while congruent information increased labelling agreement. We initially tested congruent and incongruent effects against zero in the *initial baseline model* with alternative parameterisation. This showed congruent and incongruent effects both differed significantly from zero in divergent directions. Congruent effects increased agreement, *g*_*av*_ = 1.03 versus 0, *t*(22.45) = 6.27, *p* < .001, and incongruent effects decreased agreement *g*_*av*_ = −2.49 versus 0, *t*(15.3) = −6.28, *p* < .001. This finding confirmed our decision to reverse-score the incongruent effects for the remaining meta-analytic models.

Critically, the effects from incongruent information (mean change in extracted data of −29.1% vs. 12.6%) were significantly larger than from congruent information, evidenced by a significant main effect of congruency in the *main model*, *incongruent g*_*av*_ = 3.23 versus *congruent g*_*av*_ = 0.70, *F*(3, 5.22) = 10.38, *p* = .012 (see Table 1 for model fit statistics). Note, the larger effect of incongruent information was still evident after excluding incongruent emotion pairings that were significantly larger than other pairings, *F*(3, 4.11) = 13.88, *p* = .013 (see Online Supplementary Material 10 for analyses). This finding indicates that the larger effect size for incongruent than congruent pairings was not driven by a few specific pairings, and supports the robustness of the overall result.

#### Do effect sizes differ for the effect of faces and the effect of context?

Overall, we found strong evidence for both the *effect of faces* and the *effect of context* (mean change in extracted data was 30.9% and 14.2%, respectively). Both effect types differed significantly from zero, *effect of faces*: *g*_*av*_ = 3.23 vs. 0, *F*(2, 9.05) = 7.24, *p* = .013; *effect of context*: *g*_*av*_ = 1.23 vs. 0, *F*(2, 17.29) = 14.50, *p* < .001. However, while the effect of faces was numerically larger than the effect of context, this difference was not statistically significant when congruency and clarity-difference effects were accounted for in the *main model*. Specifically, omnibus Wald-type tests did not find a significant main effect of effect type, *F*(3, 6.31) = 1.86, *p* = .232. There was no evidence this pattern varied by other moderators, as indicated by the non-significance effect type interactions in the model, *p*s > .096.

#### Do relatively clearer stimuli influence labelling agreement more?

**Fig. 5:**
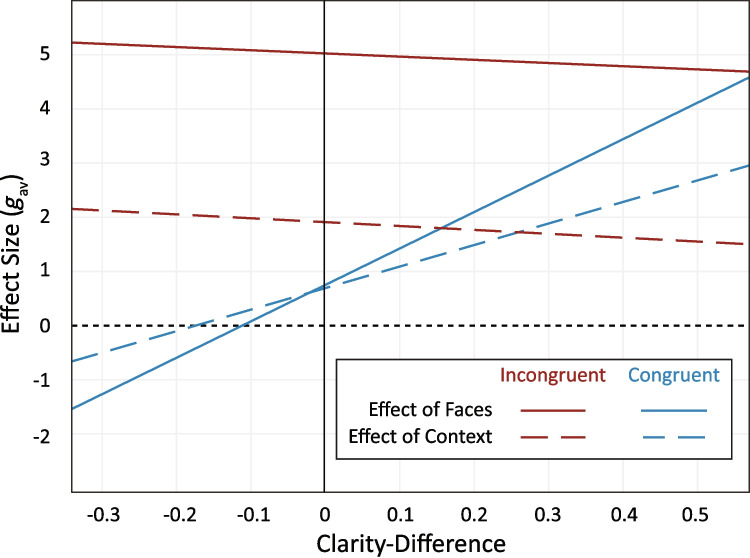
**Interaction between congruency and clarity-difference scores***.* Clarity-difference represents the difference in proportion agreement between the added stimulus in isolation minus the agreement of the target stimulus in isolation. Positive clarity-difference scores reflect a clearer added stimulus, whereas negative clarity-difference scores reflect a clearer target stimulus. Zero reflects no difference in clarity between target and added stimulus

Figure 5 shows adding stimuli with greater clarity relative to the target stimuli increased effect sizes for congruent but not incongruent pairings. While the main effect for clarity-difference was significant, *F*(3, 3.23) = 47.96, *p* = .004, this was subject to a significant clarity-difference*congruency interaction, *t*(4.71) = −2.86, *p* = .038. Follow-up post hoc comparisons revealed congruent effect sizes increased with higher clarity-difference scores, *no clarity-difference g*_*av*_ = 0.70 versus *clarity-difference of 0.01 g*_*av*_ = 0.75, *t*(6.13) = 12.23, *p* < .001. Contrastingly, there was no significant change in incongruent effects with clarity-difference scores, *no clarity-difference g*_*av*_ = 3.23 versus *clarity-difference of 0.01 g*_*av*_ = 3.23, *t*(6.36) = −0.68, *p* = .522.^3^ That is, when faces and visual context are congruent, adding relatively clearer emotion signals results in a larger change to perceptual agreement. However, when faces and visual context are incongruent, the relative clarity of the emotion signals does not affect the change to perceptual agreement.

To further investigate the separate contributions of the target and added stimuli clarity, we reran the model using absolute-clarity scores in place of clarity-difference scores. We did this separately for the absolute-clarity scores of the target (*target-absolute-clarity*) and the added stimulus (*added-absolute-clarity*) as there were insufficient data to include both in the same model. That is, including both types of absolute-clarity scores led to comparison groups with too few observations to adequately test the whole model. The results revealed that the contributions of clarity come primarily from the target, with the results from the model including target-absolute-clarity aligning with the results of the main model. Specifically, the analyses revealed significant main effects for both target-absolute-clarity, *F*(3, 6.47) = 4.96, *p* = .042, and congruency, *F*(3, 6.56) = 14.62, *p* = .003, as well as a significant interaction between them, *t*(7.79) = 3.10, *p* = .015. Post hoc analyses found congruent effects decreased^4^ as target-absolute-clarity increased, *target-absolute-clarity of 0 g*_*av*_ = 4.77 versus *target-absolute-clarity of 0.01 g*_*av*_ = 4.72, *t*(11.06) = −3.28, *p* = .007, whereas there was no significant effect of target-absolute-clarity for incongruent effects, *t*(5.28) = 0.63, *p* = .555. In contrast, in the added-absolute-clarity model, both the main effect of added-absolute-clarity and the added-absolute-clarity*congruency interaction were non-significant (both *p*s > .279). Using clarity-difference scores modelled the data better (Q = 16,340 vs. 17,091 for target-absolute-clarity) and was justified by our a priori rationale to model the contributions from both faces and visual context. It is also difficult to rule out any effect of added-absolute-clarity, as the lack of significance may reflect the reduced power of this model. We therefore retained the main model in the main article and report the full results of the additional absolute-clarity models in Online Supplementary Material 5 (Table S5b).

#### Do effect sizes differ by emotion category, presentation duration, or presentation format?

There was no significant main effect for any of the remaining moderators and thus these results are reported in Online Supplementary Material 5 (Table S5a).

## Discussion

The present study found the notion of facial primacy in emotion perception was not supported when accounting for key confounds – namely, relative signal clarity and emotional congruency. The large meta-analytic estimates provide strong evidence that faces and the surrounding visual context mutually influence the perception of emotion in one another. Initially, the effect of faces appeared to be numerically larger than the effect of context. Critically, however, these effects did not differ in size when key moderators, including relative signal clarity and emotional congruency, were modelled. Rather, relative signal clarity plays an important role in the perceptual integration of visual emotion cues, with congruency between the target and its surroundings driving the direction of the effects. Notably, 85% of individual effect sizes were in the direction predicted by congruency. The findings presented here align with the theory of constructed emotion indicating all visual information is integrated and variation in the clarity of the available cues has a crucial role in this process. Importantly, our work provides a valuable framework for interpreting past literature and planning further research to advance theoretical explanations of emotion integration.

Crucially, our work shows failing to account for signal clarity can lead to misleading evidence in favour of facial primacy. Indeed, the effect sizes without accounting for relative clarity make it look as though the effect of faces (*g*_*av*_ = 3.23) is almost three times larger than visual context (*g*_*av*_ = 1.23). This may reflect that most popular face stimuli show exaggerated and prototypical expressions, developed to produce high observer agreement (clarity) about the emotion being signalled (Dawel et al., 2021). In contrast, agreement may be lower for other types of visual context (e.g., Martinez et al., 2016). Thus, there is a confound whereby face stimuli may signal emotions more clearly than the context they are paired with. Supporting this argument, Van den Stock et al. (2007) and Karaaslan et al. (2020) both found the influence of bodies on emotion perception was larger when facial expressions were less clear. Consistent with these findings, we find that when a paired congruent stimulus, such as a face or body posture, is relatively clearer than the target stimuli, it has a greater influence on emotion labelling.

Overall, our results highlight the need to systematically investigate the role of signal clarity in integrative emotion perception. At a minimum, future studies should control for signal clarity. Ideally, however, experimental manipulation and explicit modelling of signal clarity could reveal how people integrate emotion signals that differ in clarity levels. Within this line of work, there is also a need to further consider how clarity should be captured. Here, we were bound by the available data, which enabled us to model these effects across a large number of experiments for the first time. While our findings indicate observer agreement can be a useful measure of signal clarity, alternative operationalisations should be considered. Experimentally, for example, factors such as expression intensity (Vikhanova et al., 2022) and image quality (Kinchella & Guo, 2021) could be manipulated to investigate the role of clarity in emotion perception.

Interestingly, we found higher relative clarity enhanced agreement for congruent but not incongruent pairings. Semantic network theory may help explain this finding. Recall that semantic network theory suggests information is represented by nodes that are interconnected within clusters of related information. Within this model, congruent cues would facilitate emotion perception by co-activating nodes (and the connections between them) within a single emotion cluster (Berthet et al., 2011; Marko & Riečanský, 2021). For instance, adding clenched fists to an irritated face would increase activation within the anger cluster, facilitating labelling. The less clear the target is, the greater the change in co-activation when new information is added, resulting in a greater increase in labelling agreement. In contrast, incongruent cues activate clusters for other emotions. By activating other emotion clusters, incongruent cues impede labelling. However, we did not observe a relationship between clarity and incongruent effect size, which may reflect the role of clarity is not straightforward when emotion pairings are mismatched. Applying semantic network theory to emotion perception in this way provides a framework for investigating the integration of emotion signals from other cues, such as auditory stimuli or social vignettes (Gerdes et al., 2014; Krumhuber et al., 2023). Understanding how we integrate these broader emotion signals during emotion perception is critical for explaining how we interpret others’ emotional states in our daily lives.

The consistently large effect of incongruent cues likely reflects people’s high sensitivity to unexpected information. Experiencing unexpected information leads to greater neural activity relative to expected information (De Lange et al., 2018; Peelen et al., 2024; Press et al., 2020; Steinhauser & Hübner, 2009; Wendelken et al., 2009). This increased activity, known as a prediction error, is thought to act as feedback to update expectations relative to actual experiences (Press et al., 2020). For example, if we see a face signalling anger, we expect that person to be feeling angry. However, if their body signals sadness then prediction error would prompt us to adjust our judgement to incorporate sadness, not just anger. This sensitivity to unexpected or incongruent information helps us understand our findings of large incongruent effects. While prediction error provides a parsimonious explanation of the large effects of incongruency, the smaller size of congruency effects may also reflect that there is limited scope for them to manifest, due to the labelling of isolated stimuli often being close to ceiling. This ceiling effect means that there would be less room for congruent stimuli to increase emotion labelling agreement than for incongruent stimuli to decrease agreement. These effects are important to understand because we learn how to respond to uncertain situations through others’ emotional signals (Sorce et al., 1985). If the cues we observe are incongruent and make the intended emotion less apparent, we will be slower to learn the appropriate response to a situation. This is particularly prescient when we consider fear, a helpful emotion to identify quickly, is more regularly displayed in incongruent pairings, with real (i.e., non-posed) facial expressions of fear more often identified as surprise than as fear (Abramson et al., 2017). Understanding the integration of incongruent information will be pertinent for explaining how we learn and/or misinterpret information from others (Hess et al., 2013).

## Limitations and future directions

This work paves the way for several important lines of future investigation. First, while our findings are limited to the integration of visual stimuli, our approach provides a framework for testing generalisability across sensory domains (e.g., auditory stimuli; Gerdes et al., 2014; Vesker et al., 2018) or for top-down processing (e.g., social context; Krumhuber et al., 2023). If further investigations show consistency across different types of affective stimuli, our results may set a foundation for a generalised understanding of emotion integration across inputs. Second, our models retained significant residual heterogeneity, highlighting our analyses do not fully explain the *effect of faces* or the *effect of context.* Thus, there is a need to investigate additional moderators. Our focus on study-level moderators opens the door for research to test if individual differences, such as social anxiety (Lynn et al., 2018) or empathy (Olderbak & Wilhelm, 2017), might explain some of this variance. This line of inquiry has potential to reveal if and how emotion cue integration contributes to clinical differences in emotion perception (Kumfor et al., 2018). Third, it would be valuable to further investigate the childhood developmental emergence of face-context integration. All four studies that included participants under the age of 18 years showed children integrate emotion signals (Malaia et al., 2019; Nelson & Mondloch, 2017; Nelson & Russell, 2012; Nguyen & Nelson, 2021). However, while the trajectory of integration has previously been investigated (Theurel et al., 2016), it is currently unclear when the capacity to integrate emotion signals fully matures. It is possible, for example, that facial primacy is present before children develop basic facial emotion discrimination, around 4 years of age (Widen, 2013), but is lost once emotion integration matures. Fourth, future investigations should further explore the unique contributions of faces and visual context. Whilst our analyses provide valuable evidence for which cue is more influential in altering labelling, it cannot speak to which cue contributes most during emotion perception. Although a few studies have started to investigate the unique contribution of cues (e.g., Chen & Whitney, 2019, 2020, 2021; Lecker et al., 2020; Reschke & Walle, 2021), further understanding the unique contributions of different emotional cues is pivotal for the field. Finally, our findings need to be considered in light of potential publication bias, which may have inflated effect sizes such that they may not be as large as we found here.

## Conclusion

The findings of this study show people’s perception of others’ emotional states is not driven by facial primacy (i.e., faces more than context) once important moderators – stimulus congruency and relative stimulus clarity – are accounted for. We show our perception of emotions is integrative, incorporating information from both faces and context. Importantly, the content and clarity of the emotion signal is more important than the signal source. It is pertinent for the literature to consider the larger influence of incongruent emotional signals as it will provide valuable insight into how we understand others, as emotional displays are not always congruent. However, it is also vital the literature consider the influence of signal clarity in future investigations of emotion perception, so as not to confound the effects of signal clarity with facial primacy. The world is inherently ambiguous; failing to adequately consider the clarity of the stimuli we are using risks missing the real-world human perceptual experience.

## Supplementary Information

Below is the link to the electronic supplementary material.

Supplementary file1 (DOCX 1.08 MB)

## Data Availability

The protocol and datasets generated and analysed during the current study are available in the OSF repository at: https://osf.io/mn7qk/.

## Footnotes

1 This conservative decision was made as we could not identify the exact cause as to why the studentised residuals could not be calculated. We note that the metafor documentation indicates that “It may not be possible to fit the model after deletion of the *i*th case from the dataset. This will result in NA values for that case” (https://wviechtb.github.io/metafor/reference/residuals.rma.html).

2 Note, we pre-registered separate models for the *effect of context* and the *effect of faces* but opted to combine them into one model because it allowed us to directly compare the size of these effects and test their relationships with moderator variables.

3 A clarity-difference score of 0.01 was used to calculate the effect size estimates as it reflects a 1% increase in clarity-difference (which ranges between -1 and 1). This is consistent with general reporting of continuous variables on a 100-point scale in regression models.

4 Target-absolute-clarity effect sizes are in the opposite direction to clarity-difference effects because larger clarity-difference scores occur when targets have increasingly lower absolute-clarity than added stimuli (i.e., clarity-difference = added-clarity *minus* target-clarity).

## Code availability

The coding used for analyses are available in the OSF repository at: https://osf.io/mn7qk/.

## Declarations

### Open practices statement

The protocol for this systematic review and meta-analysis was preregistered on 2 May 2022. The protocol and datasets generated and analysed during the current study are available in the OSF repository at: https://osf.io/mn7qk/.
