# Supplementary material for: Interactions between faces and visual context in emotion perception: A meta-analysis
Source: Psychon Bull Rev. 2025 Apr 3;32(5):1987–2003. doi: 10.3758/s13423-025-02678-6 (PMC12426097; doi:10.3758/s13423-025-02678-6)
Supplement: Supplementary file 1 — Supplementary file1 (DOCX 1.08 MB) [file 13423_2025_2678_MOESM1_ESM.docx]

# Supplemental Materials

[Supplement 1: Search Syntax 2](#_Toc190193868)

[Supplement 2: Summary of Articles Included in the Meta-Analyses 3](#_Toc190193869)

[Supplement 3: Statistical Processes 4](#_Toc190193870)

[Supplement 4: Comparison Between Two-Level and Three-Level Model Fit Statistics 5](#_Toc190193871)

[Supplement 5: Meta-Regressions of Pooled Labelling Agreement Data 6](#_Toc190193872)

[Supplement 6: Outliers and Influential Statistics 9](#_Toc190193873)

[Supplement 7: Evaluation of GRADE Criteria 13](#_Toc190193874)

[Supplement 8: Rating Models 15](#_Toc190193875)

[Supplement 9: Reaction Time Models 17](#_Toc190193876)

[Supplement 10: Incongruent Effects Across Emotion Pairings 19](#_Toc190193877)

# Supplement 1: Search Syntax

Search Term Syntax

PsycInfo:

#1: ((((emotion* or valenc*) adj3 (perception or identif* or label* or recogni* or judg* or content)) or (valenc* adj3 (positiv* or negativ*))) and (face or faces or facial or expression*) and (context* or "body language" or posture or pose or gesture or scene or (background adj3 (emotion* or valenc*)))).tw.

#2: limit 1 to (english language and peer reviewed journal)

Scopus:

#1 TITLE-ABS-KEY ( ( ( emotion* OR valenc* ) W/3 ( perception OR identif* OR label* OR recogni* OR judg* OR content ) ) OR ( valenc* W/3 ( positiv* OR negativ* ) ) ) AND TITLE-ABS-KEY ( face OR faces OR facial OR expression* ) AND TITLE-ABS-KEY ( context* OR "body language" OR posture OR pose OR gesture OR scene OR ( background W/3 ( emotion* OR valenc* ) ) ) AND ( LIMIT-TO ( LANGUAGE , "English" ) ) AND ( LIMIT-TO ( SRCTYPE , "j" ) )

Web of Science:

#1 TS=(((( emotion* or valenc*) NEAR/3 ( perception OR identif* OR label* OR recogni* OR judg* OR content )) OR (valenc* NEAR/3 (positiv* OR negativ*))) AND (face OR faces OR facial OR expression*) AND (context* OR "body language" OR posture OR pose OR gesture OR scene OR (background NEAR/3 ( emotion* or valenc*))))

#2 Refined By: Languages: English

#3 Refined By: Document Types: Articles or Review Articles

# Supplement 2: Summary of Articles Included in the Meta-Analyses

## Table S2

*Articles Included in the Meta-Analyses of Labelling Effects*

| **Author(s) and year** | **Observers** | **Effects (*n*)** |
| --- | --- | --- |
| Abramson et al. (2017) | 323 | 36 |
| Aviezer et al. (2009) | 27 | 4 |
| Aviezer, Hassin, and Bentin (2012) | 7 | 9 |
| Aviezer, Trope, and Todorov (2012b) | 47 | 22 |
| Bjornsdottir et al. (2017) | 214 | 96 |
| Brewer et al. (2017) | 27 | 12 |
| Civile and Obhi (2016) | 24 | 4 |
| Foul et al. (2018) | 56 | 63 |
| Foul et al. (2022) | 238 | 40 |
| Hümmer et al. (2021) | 30 | 6 |
| Kret et al. (2013) | 37 | 18 |
| Lecker and Aviezer (2021) | 277 | 18 |
| Lecker et al. (2020) | 123 | 64 |
| Lee and Van Meter (2020) | 29 | 5 |
| Li (2021) | 28 | 4 |
| Malaia et al. (2019) | 14 | 2 |
| Martinez et al. (2016) | 92 | 72 |
| Meeren et al. (2005) | 12 | 4 |
| Minton and Mienaltowski (2021) | 93 | 28 |
| Nelson and Mondloch (2017) | 80 | 48 |
| Nelson and Russell (2012) | 108 | 36 |
| Nguyen and Nelson (2021) | 90 | 12 |
| Noh and Isaacowitz (2013) | 84 | 16 |
| Perry et al. (2013) | 30 | 4 |
| Pollux et al. (2019) | 65 | 10 |
| Sasson et al. (2016) | 39 | 85 |
| Van Der Zant and Nelson (2021) | 132 | 8 |
| Visch et al. (2014) | 31 | 4 |
| Willis et al. (2011) | 31 | 18 |
| Xu et al. (2017) | 15 | 4 |

# Supplement 3: Statistical Processes

## Effect Size Calculations

Following the recommendation of Lakens (2013), we opted to use Hedge’s *g_av_* because the majority of articles in the meta-analysis used within-participant designs.

We first calculated Cohen’s *d*_av_:

$$Cohen^{'}sd_{av}=\frac{\mu_{diff}}{\begin{aligned} \frac{{SD}_{iso}+{SD}_{comb}}{2} \end{aligned}}$$

And then applied the Hedge’s *g* correction:

$$Hedge'sg_{av}=d_{av}*\left( 1-\left( \frac{3}{4*n-1}-1 \right) \right)$$

# Supplement 4: Comparison Between Two-Level and Three-Level Model Fit Statistics

## Table S4

*Model Fit Statistics for Two- and Three-Level Models of Labelling Agreement Effects*

| **Model** | ***g_av_* [95% CI]** | **SE** | ***t*** | ***p*** | **Q** | **I^2^_(2)_** | **I^2^_(3)_** | **LRT** |
| --- | --- | --- | --- | --- | --- | --- | --- | --- |
| *Initial Baseline Model* | | | | | | | | |
| Two-level (*n* = 752) | -0.90 [-2.23, 0.42] | 0.62 | -1.47 | .165 | 33,900 |  |  |  |
| Three-level (*k* = 30, *n* = 752) | -0.48 [-1.14, 0.18] | 0.32 | -1.50 | .146 | 33,900 | 0.21 | 0.78 | χ^2^ (1) = 213.39  *p* < .001 |
| *Score-Adjusted Baseline* | | | | | | | | |
| Two-level  (*n* = 752) | 2.09 [1.47, 2.72] | 0.29 | 7.21 | < .001 | 23,451 |  |  |  |
| Three-level  (*k* = 30, *n* = 752) | 1.66 [1.18, 2.14] | 0.23 | 7.15 | < .001 | 23,451 | 0.12 | 0.87 | χ^2^ (1) = 63.50  *p* < .001 |

# **Supplement 5:** Meta-Regressions of Pooled Labelling Agreement Data

## Table S5a

*Meta-Regressions of Moderator Effects for Labelling Agreement Models*

|  | **Vs reference level** | | | | **Wald Omnibus** | |
| --- | --- | --- | --- | --- | --- | --- |
| **Model** | ***g_av_* [95% CI]** | **SE** | ***t*** | ***p*** | **F** | ***p*** |
| **Reduced Model** (*k* = 30, *n* = 752)  I^2^_(2)_/ I^2^_(3)_ = 0.15/0.83; Q = 21,266; LRT = χ^2^(3) = 221.1, *p* < .001 | | | | | | |
| Intercept | 0.62 [0.05, 1.19] | 0.28 | 2.25 | .034 |  |  |
| Effect of Faces (vs ref = Effect of context) | 0.58 [-1.12, 2.29] | 0.75 | 0.78 | .458 | 0.60 | .458 |
| **Incongruent (vs ref = congruent effects)** | **1.22 [0.50, 1.94]** | **0.33** | **3.65** | **.003** | **13.30** | **.003** |
| Congruency*Effect Type | 2.84 [-0.87, 6.55] | 1.64 | 1.73 | .118 |  |  |
| **Clarity-Difference Model + Isolated Stimulus Emotion Category** (*k* = 21, *n* = 589)  I^2^_(2)_/ I^2^_(3)_ = 0.17/0.82; Q = 13,651; LRT = χ^2^(4) = 11.71, *p* = .020 | | | | | | |
| Intercept | 0.41 [-0.34, 1.17] | 0.36 | 1.16 | .262 |  |  |
| Effect of Faces (vs ref = Effect of context) | 0.13 [-0.18, 0.44] | 0.13 | 0.97 | .361 | 1.51 | .322 |
| **Incongruent (vs ref = congruent effects)** | **1.38 [0.59, 2.17]** | **0.36** | **3.86** | **.003** | **10.50** | **.016** |
| **Clarity-Difference (vs ref = 0)** | **4.86 [3.66, 6.06]** | **0.46** | **10.55** | **< .001** | **27.34** | **.024** |
| **Clarity-Difference*Congruency** | **-5.86 [-11.43, -0.28]** | **2.04** | **-2.88** | **.043** |  |  |
| Clarity-Difference*Effect Type | 0.99 [-3.01, 4.98] | 1.64 | 0.60 | .570 |  |  |
| Congruency*Effect Type | 3.09 [-0.73, 6.91] | 1.65 | 1.88 | .099 |  |  |
| *Emotions* (vs ref = Anger) |  |  |  |  | 1.32 | .360 |
| Disgust | 0.25 [-0.62, 1.13] | 0.38 | 0.66 | .525 |  |  |
| Fear | -0.12 [-0.48, 0.24] | 0.16 | -0.73 | .482 |  |  |
| Happiness | 0.89 [-0.61, 2.40] | 0.65 | 1.37 | .209 |  |  |
| Sadness | 0.31 [-0.46, 1.08] | 0.34 | 0.92 | .382 |  |  |
| **Clarity-Difference Model + Added Stimulus Emotion Category** (*k* = 21, *n* = 591)  I^2^_(2)_/ I^2^_(3)_ = 0.19/0.80; Q = 13,661; LRT = χ^2^(4) = 4.59, *p* = .331 | | | | | | |
| **Intercept** | **0.84 [0.23, 1.44]** | **0.29** | **2.91** | **.010** |  |  |
| Effect of Faces (vs ref = Effect of context) | 0.14 [-0.15, 0.43] | 0.13 | 1.10 | .305 | 1.81 | .263 |
| **Incongruent (vs ref = congruent effects)** | **1.46 [0.75, 2.16]** | **0.32** | **4.59** | **< .001** | **10.35** | **.017** |
| **Clarity-Difference (vs ref = 0)** | **4.29 [3.51, 5.08]** | **0.30** | **14.26** | **< .001** | **42.37** | **.013** |
| Clarity-Difference*Effect Type | 2.10 [-1.38, 5.59] | 1.44 | 1.46 | .192 |  |  |
| Congruency*Effect Type | 2.83 [-0.48, 6.14] | 1.43 | 1.98 | .084 |  |  |
| **Clarity-Difference*Congruency** | **-5.94 [-11.46, -0.41]** | **2.01** | **-2.95** | **.040** |  |  |
| *Emotions* (vs ref = Anger) |  |  |  |  | 4.13 | .059 |
| Disgust | -0.13 [-0.62, 0.35] | 0.21 | -0.63 | .544 |  |  |
| Fear | -0.31 [-0.77, 0.16] | 0.21 | -1.47 | .171 |  |  |
| Happiness | 0.02 [-0.77, 0.81] | 0.33 | 0.06 | .954 |  |  |
| **Sadness** | **-0.52 [-0.89, -0.15]** | **0.16** | **-3.23** | **.012** |  |  |
| **Clarity-Difference Model + Presentation Length (limited vs unlimited)** (*k* = 22, *n* = 557)  I^2^_(2)_/ I^2^_(3)_ = 0.23/0.76; Q = 13,086; LRT = χ^2^(1) = 0.11, *p* = .738 | | | | | | |
| Intercept | 0.57 [-0.19, 1.33] | 0.34 | 1.65 | .130 |  |  |
| Effect of Faces (vs ref = Effect of context) | 0.12 [-0.26, 0.49] | 0.17 | 0.70 | .498 | 1.77 | .267 |
| **Incongruent (vs ref = congruent effects)** | **1.53 [0.63, 2.44]** | **0.40** | **3.84** | **.004** | **17.27** | **.006** |
| **Clarity-Difference (vs ref = 0)** | **4.40 [3.17, 5.64]** | **0.50** | **8.88** | **< .001** | **20.78** | **.010** |
| Clarity-Difference*Effect Type | 1.34 [-3.63, 6.31] | 2.02 | 0.66 | .532 |  |  |
| Congruency*Effect Type | 3.58 [-0.75, 7.92] | 1.85 | 1.94 | .092 |  |  |
| **Clarity-Difference*Congruency** | **-9.73 [-15.58, -3.88]** | **2.29** | **-4.26** | **.008** |  |  |
| Unlimited Presentation | 0.20 [-0.82, 1.22] | 0.46 | 0.44 | .672 | 0.19 | .672 |
| **Clarity-Difference Model + Limited Presentation Length (ms)** (*k* = 10, *n* = 225)  I^2^_(2)_/ I^2^_(3)_ = 0.39/0.59; Q = 3,272; LRT = χ^2^(1) = 1.15, *p* = .284 | | | | | | |
| Intercept | 0.35 [-0.41, 1.11] | 0.31 | 1.11 | .306 |  |  |
| Effect of Faces (vs ref = Effect of context) | -0.31 [-0.93, 0.31] | 0.20 | -1.53 | .216 | 0.41 | .800 |
| Incongruent (vs ref = congruent effects) | 1.57 [-0.57, 3.72] | 0.68 | 2.30 | .103 | 3.43 | .512 |
| Clarity-Difference (vs ref = 0) | 3.94 [1.90, 5.99] | 0.41 | 9.69 | .017 | 9.26 | .378 |
| Clarity-Difference*Effect Type | 4.02 [-11.19, 19.23] | 2.68 | 1.50 | .304 |  |  |
| Congruency*Effect Type | 2.46 [4.71, 9.64] | 2.06 | 1.20 | .329 |  |  |
| Clarity-Difference*Congruency | -10.80 [-25.80, 4.19] | 4.24 | -2.55 | .099 |  |  |
| Presentation Time (ms) | 0.00 [-0.00, 0.00] | 0.00 | 1.00 | .460 | 1.01 | .460 |
| **Clarity-Difference Model + Presentation Format (Static vs Dynamic)** (*k* = 24, *n* = 701)  I^2^_(2)_/ I^2^_(3)_ = 0.15/0.83; Q = 15,447; LRT = χ^2^(1) = 3.02, *p* = .084 | | | | | | |
| **Intercept** | **0.53 [0.11, 0.95]** | **0.20** | **2.66** | **.016** |  |  |
| Effect of Faces | 0.03 [-0.24, 0.29] | 0.12 | 0.21 | .834 | 1.90 | .226 |
| **Incongruent** | **1.34 [0.55, 2.13]** | **0.36** | **3.74** | **.003** | **10.43** | **.012** |
| **Clarity-Difference** | **4.48 [3.66, 5.30]** | **0.34** | **13.29** | **< .001** | **49.59** | **.003** |
| Clarity-Difference*Effect Type | 1.56 [-1.61, 4.73] | 1.36 | 1.14 | .289 |  |  |
| Congruency*Effect Type | 3.07 [-0.68, 6.82] | 1.64 | 1.87 | .097 |  |  |
| **Clarity-Difference*Congruency** | **-5.83 [-11.22, -0.45]** | **2.05** | **-2.84** | **.039** |  |  |
| Dynamic | 0.70 [-0.40, 1.80] | 0.35 | 1.98 | .138 | 3.91 | .138 |

*Note.* Reference levels for the intercept are effect type = effect of context, congruency = congruent effects, clarity-difference = 0, emotion category = anger, presentation length (unlimited vs limited) = limited, presentation length (ms) = 0ms, presentation format = static. LRT = Likelihood Ratio Test. Wald omnibus = estimated main effect of factor using supplemental omnibus Wald-type test.

## Absolute-Clarity Analyses

We ran two additional models to investigate how the absolute-clarity of the target and the added stimuli separately influence effects. We ran separate target-absolute-clarity and added-absolute-clarity models, using the absolute-clarity scores in place of clarity-difference scores. We ran these models separately as there were insufficient data to include both in the same model due to too few observations across all required comparisons.

Table S5b presents the model results. This additional modelling showed that the effects of target-absolute-clarity were consistent with those of clarity-difference scores (albeit with effect sizes in the opposite direction because larger clarity-difference scores occur when targets have increasingly lower absolute-clarity than added stimuli, i.e., clarity-difference = added- clarity *minus* target- clarity). As per the clarity-difference model, the target-absolute-clarity model showed significant main effects of target- clarity, *F*(3, 6.47) = 4.96, *p* = .042, and congruency, *F*(3, 6.56) = 14.62, *p* = .003, with a significant interaction between them, *t*(7.79) = 3.10, *p* = .015. This interaction reflected that congruent effects decreased as target-clarity increased, *t*(11.06) = -3.28, *p* = .007, whereas there was no significant effect of target-clarity for incongruent effects, *t*(5.28) = 0.63, *p* = .555.

For the added-absolute-clarity model, there continued to be a main effect of congruency, *F*(3, 5.22) = 8.84, *p* = .018, as there was in the main clarity-difference model. However, unlike for clarity-difference scores, we found that there was no significant main effect of added-clarity, *F*(3, 5.01) = 1.35, *p* = .358, and the added-clarity*congruency interaction was also non-significant, *t*(6.17) = -1.18, *p* = .280.

## Table S5b

*Additional Models Investigating Absolute Clarity Scores of Target and Added Stimuli*

|  | **Vs reference level** | | | | **Wald Omnibus** | |
| --- | --- | --- | --- | --- | --- | --- |
| **Model** | ***g_av_* [95% CI]** | **SE** | ***t*** | ***p*** | **F** | ***p*** |
| **Main Model + Target-Absolute-Clarity** (*k* = 30, *n* = 752)  I^2^_(2)_/ I^2^_(3)_ = 0.18/0.82; Q = 18,579; LRT = χ^2^(3) = 86.31, *p* < .001 | | | | | | |
| **Intercept** | **5.05 [1.94, 8.16]** | **1.40** | **3.60** | **.005** |  |  |
| Effect of faces (vs ref = Effect of context) | -0.72 [-4.73, 3.29] | 1.75 | -0.41 | .691 | 1.53 | .290 |
| Incongruent (vs ref = Congruent effects) | -4.73 [-9.64, 0.17] | 2.03 | -2.34 | .056 | **14.62** | **.003** |
| **Target Clarity (vs ref = 0)** | **-5.87 [-7.80, -1.93]** | **1.79** | **-3.28** | **.007** | **4.96** | **.042** |
| Effect Type*Congruency | 2.56 [-0.22, 5.33] | 1.21 | 2.11 | .067 |  |  |
| Effect Type* Target Clarity | 1.45 [-3.40, 6.30] | 2.19 | 0.66 | .522 |  |  |
| **Congruency* Target Clarity** | **7.50 [1.90, 13.11]** | **2.42** | **3.10** | **.015** |  |  |
| **Main Model + Added-Absolute-Clarity** (*k* = 24, *n* = 701)  I^2^_(2)_/ I^2^_(3)_ = 0.22/0.76; Q = 19,378; LRT = χ^2^(3) = 43.80, *p* < .001 | | | | | | |
| Intercept | -1.77 [-4.67, 1.12] | 1.20 | -1.47 | .188 |  |  |
| Effect of faces (vs ref = Effect of context) | -1.11 [-2.99, 0.76] | 0.82 | -1.36 | .209 | 1.47 | .312 |
| Incongruent (vs ref = Congruent effects) | 3.79 [-2.09, 9.68] | 2.42 | 1.57 | .167 | **8.84** | **.018** |
| Added Clarity (vs ref = 0) | 3.11 [-0.65, 6.88] | 1.59 | 1.96 | .092 | 1.35 | .358 |
| Effect Type*Congruency | 2.72 [-1.22, 6.67] | 1.72 | 1.58 | .151 |  |  |
| Effect Type* Added Clarity | 2.34 [-0.29, 4.96] | 1.18 | 1.99 | .076 |  |  |
| Congruency* Added Clarity | -3.03 [-9.26, 3.19] | 2.56 | -1.18 | .280 |  |  |

*Note.* Reference levels for the intercept are effect type = effect of context, congruency = congruent effects, absolute clarity = 0. LRT = Likelihood Ratio Test. Wald omnibus = estimated main effect of factor using supplemental omnibus Wald-type test.

# Supplement 6: Outliers and Influential Statistics

## Figure S6a

*Cook’s Distance at the Effect Size Level for Initially Extracted Data*


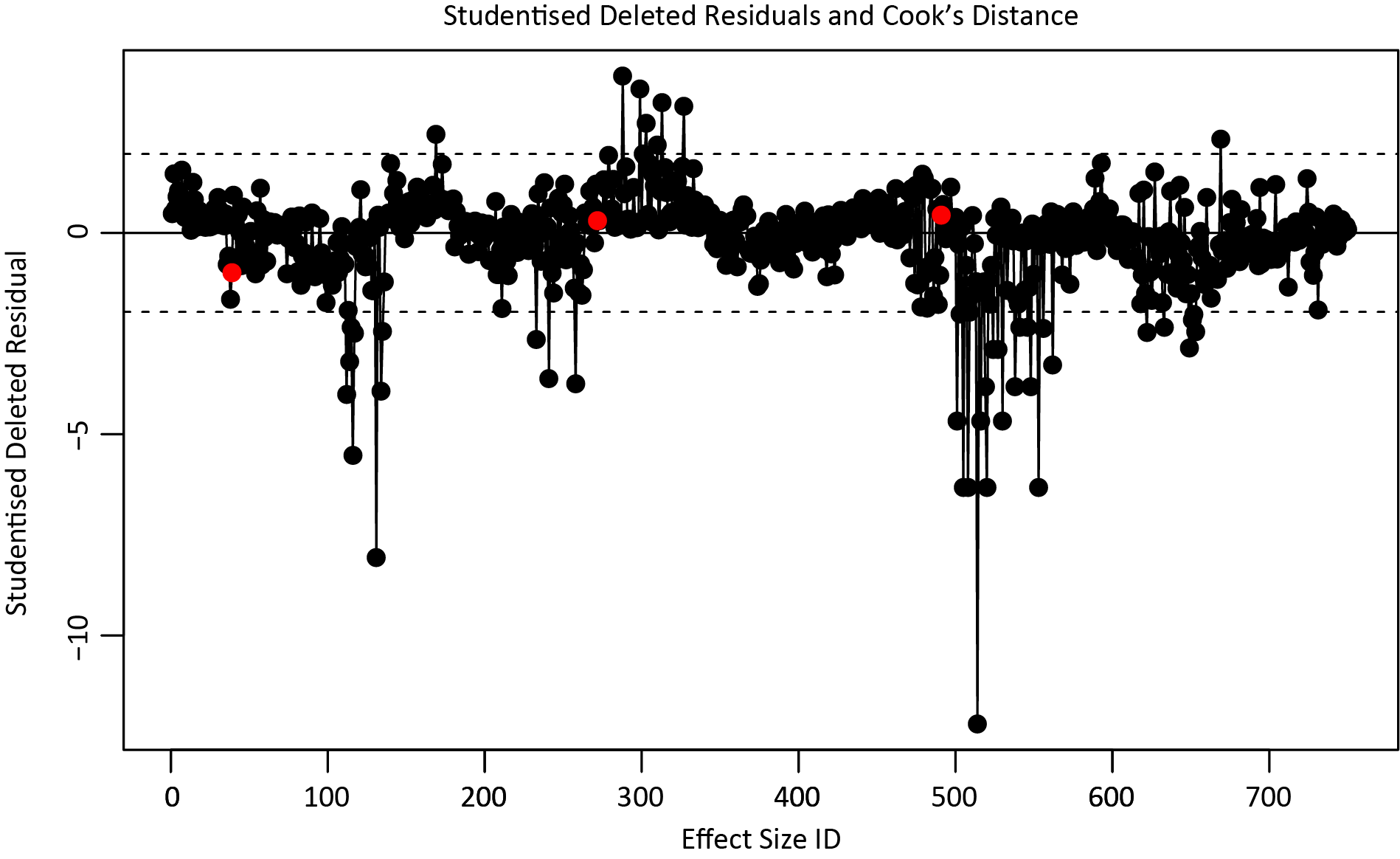


## Figure S6b


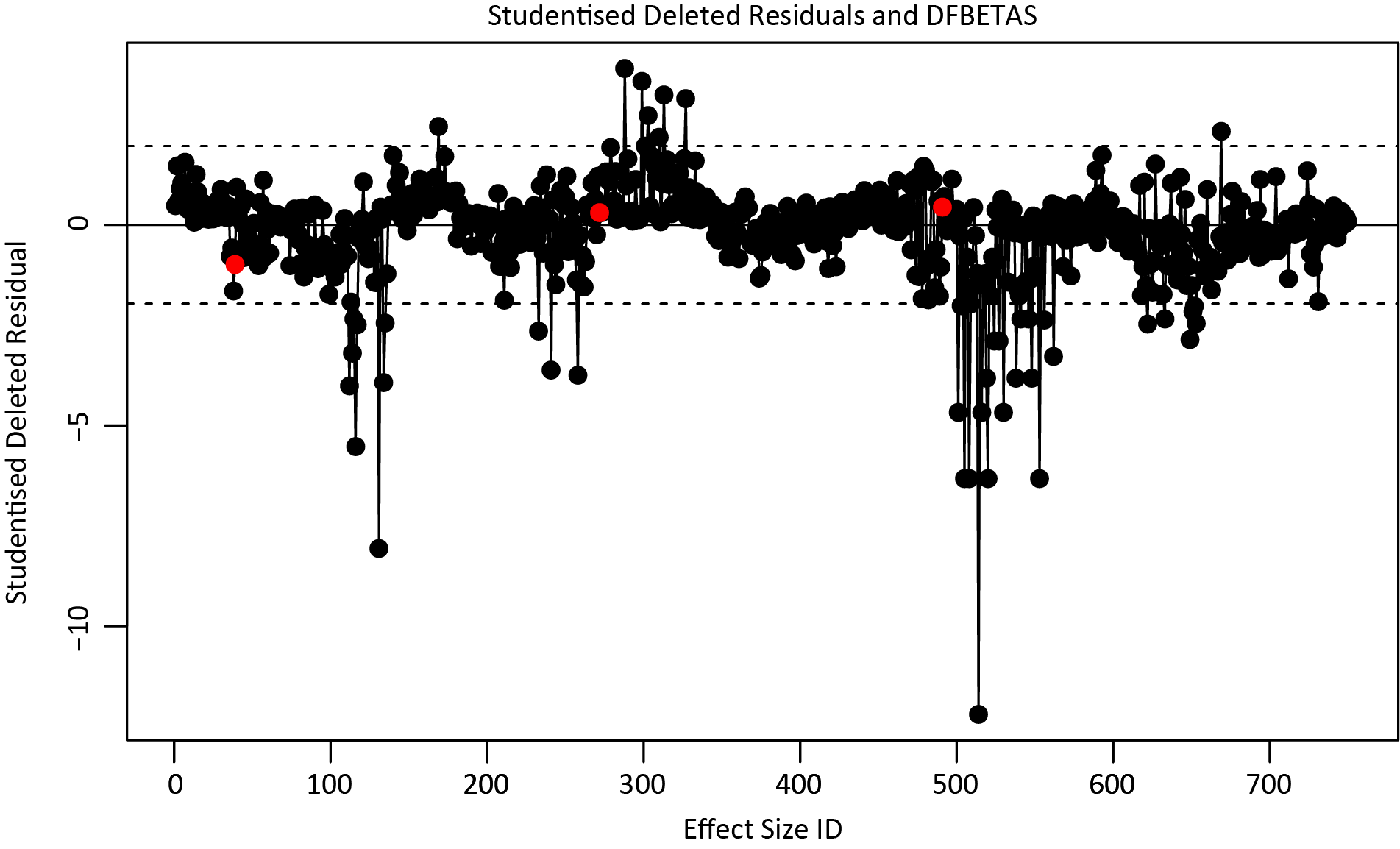
*DFBETAS at the Effect Size Level for Initially Extracted Data*

## Figure S6c

*Cook’s Distance at the Study Level for Initially Extracted Data*

##
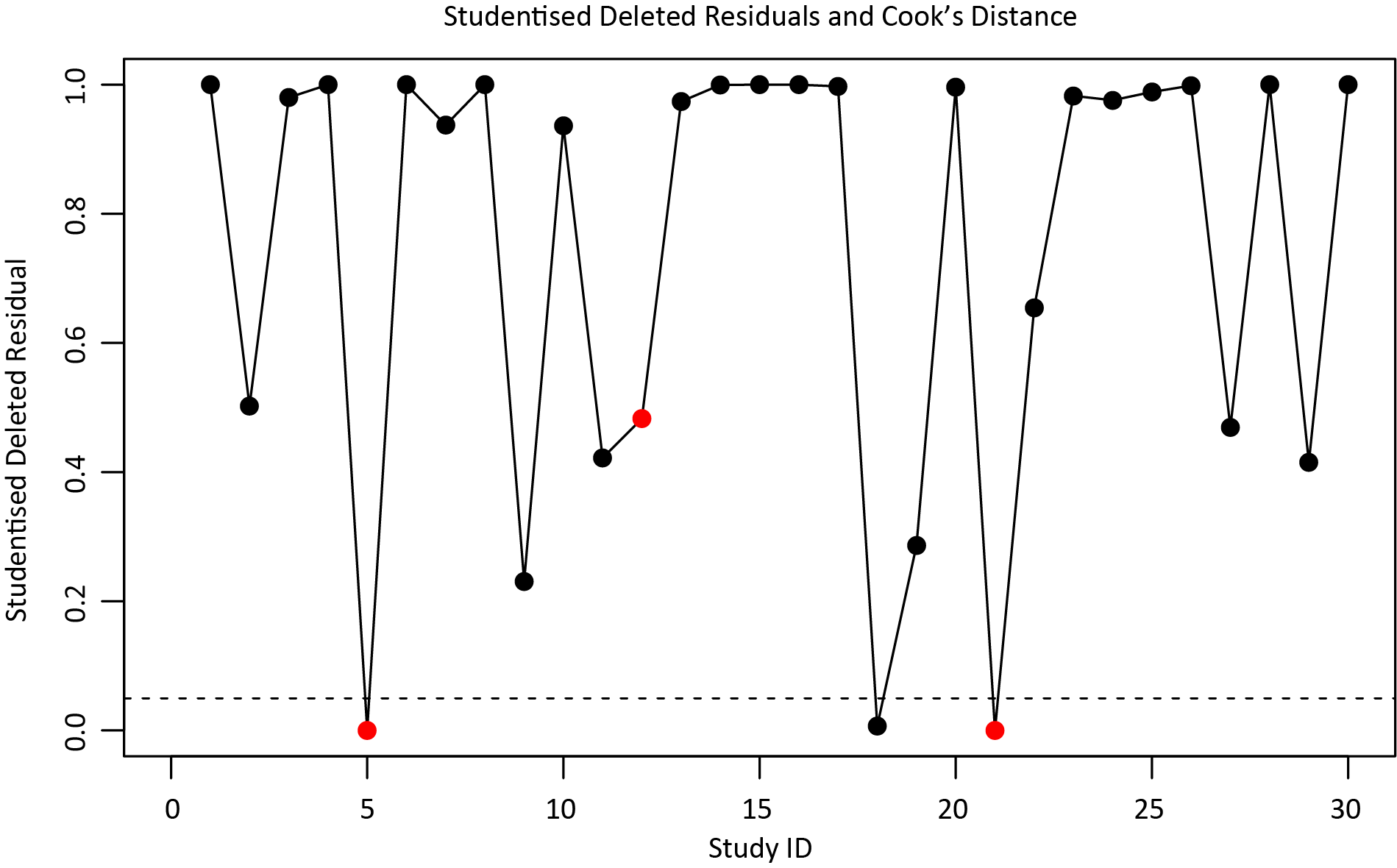
Figure S6d

*DFBETAS at the Study Level for Initially Extracted Data*
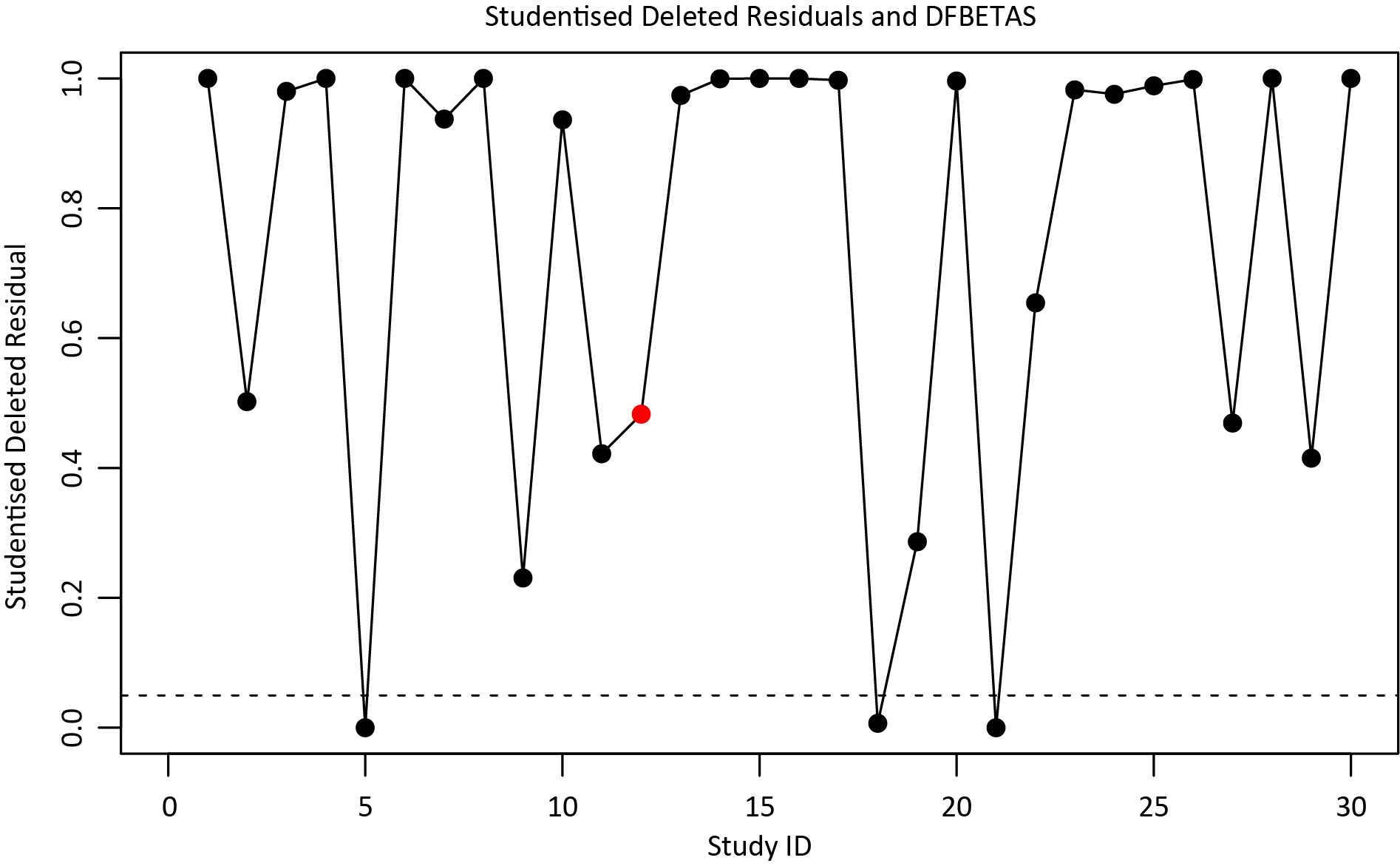


## Figure S6e

*Cook’s Distance at the Effect Size Level for Score-Adjusted Data*
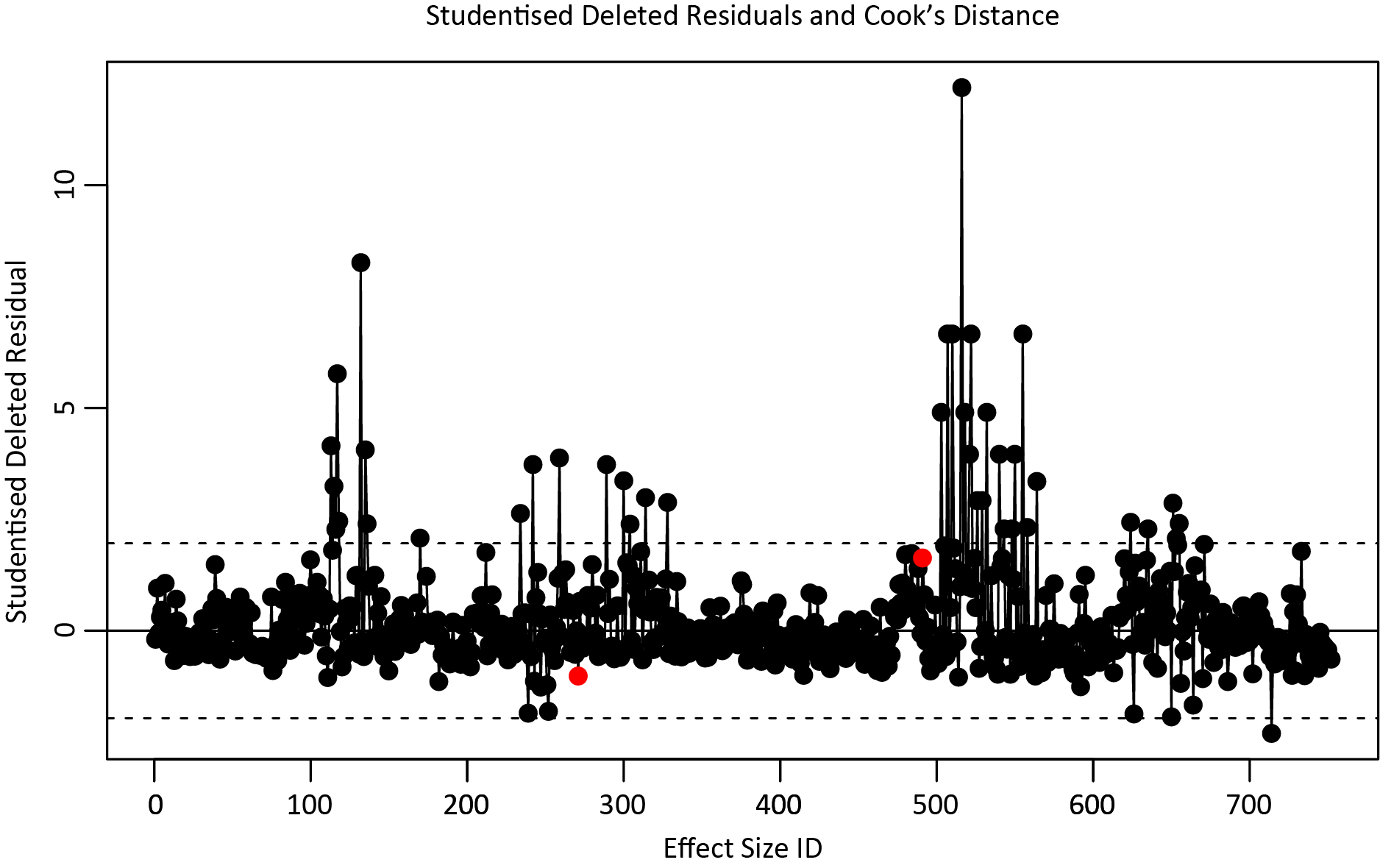


## Figure S6f

*
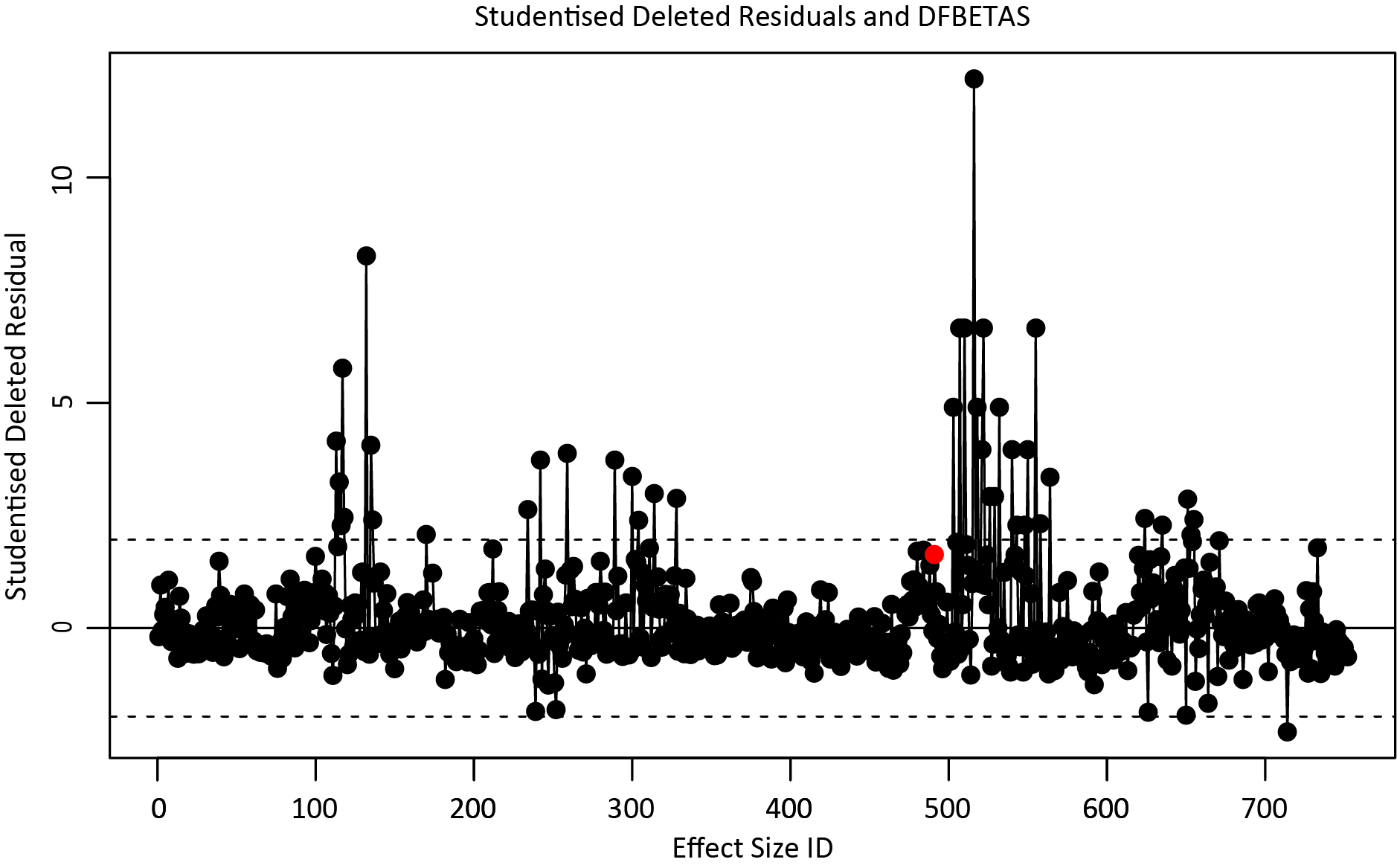
DFBETAS at the Effect Size Level for Score-Adjusted Data*

## Figure S6g

*
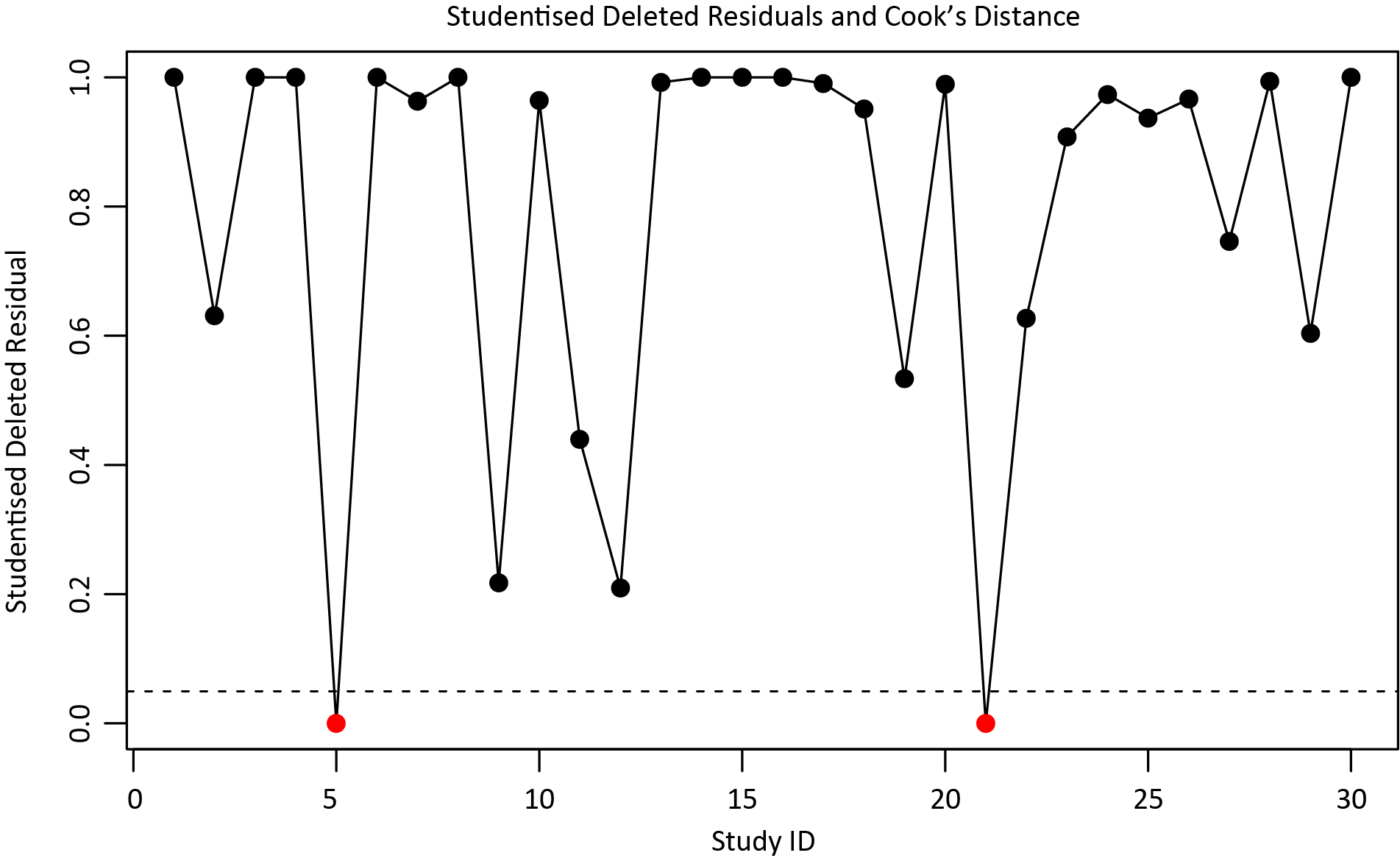
Cook’s Distance at the Study Level for Score-Adjusted Data*

## Figure S6h

*DFBETA at the Study Level for Score-Adjusted Data*


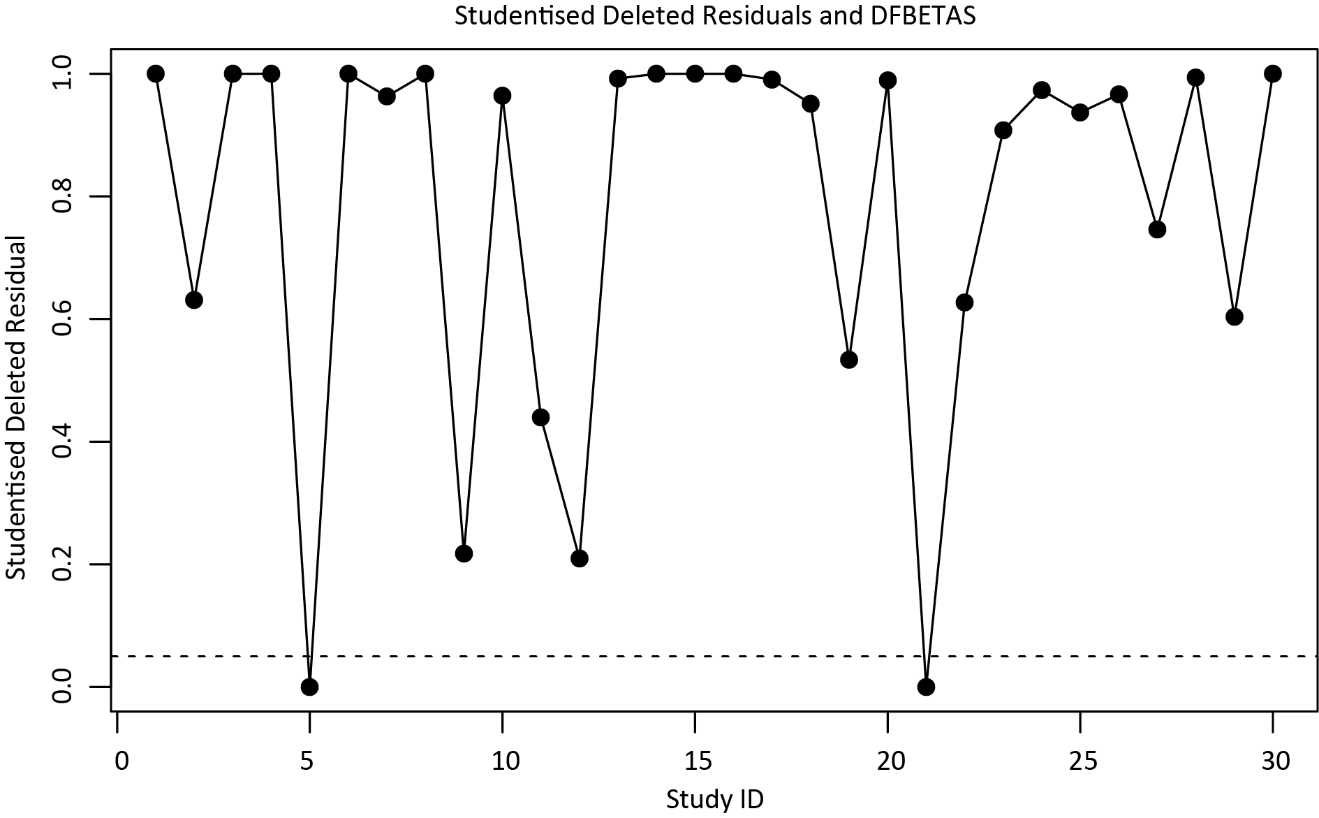


# Supplement 7: Evaluation of GRADE Criteria

Based on the framework guidelines, imprecision was assessed by considering the narrowness of estimate confidence intervals (CIs) and calculating the appropriate power and sample size for the estimated effects (known as optimal information size; OIS). Inconsistency was assessed by considering the variability of effect sizes, overlapping effect size CIs, and remaining heterogeneity after moderators were included. Publication bias was assessed by PET-PEESE, as outlined above, and how much of the eligible literature could be accessed to use in the meta-analysis. Finally, large magnitude of effect was assessed by considering the size of the effect estimates. When considered together, these criteria give a confidence rating of either, Very Low, Low, Moderate, or High.

## Imprecision

Effect sizes from the pooled data were used as a best estimate of agreement rates for isolated and paired stimuli to calculate optimal information size (OIS; α = .01, β = .90). Congruent agreement had an OIS criterion of *n* = 347, and incongruent agreement had an OIS criterion of *n* = 70. Both subsets of data reached these sample size requirements. In addition, the confidence intervals of the pooled estimate for both congruent and incongruent data did not cross zero. As such, precision of the pooled estimate can be considered adequate.

## Inconsistency

Results for congruent and incongruent agreement estimates remain consistent across the included articles with a high level of overlap in their confidence intervals. Heterogeneity assessments indicate there is high between study heterogeneity as well as significant remaining heterogeneity following a priori moderation models. However, most agreement models did significantly improve upon heterogeneity measures compared to baseline, although the remaining unexplained heterogeneity was still significant. We therefore rated down our confidence due to the high residual heterogeneity.

## Publication Bias

As highlighted in the main text, publication bias was detected through the PET-PEESE framework. Additionally, our results are based on a subset of 66% of identified articles as we were unable to access the appropriate data or results for the remaining data sources. The evidence presented is therefore biased towards available data, and is not a full representation of the published literature. As such, we again reduced confidence in the evidence.

## Large Magnitude of Effect

Both the congruent and incongruent pooled estimates are considered large by traditional cut-offs in the literature (Cohen, 1992; Funder & Ozer, 2019). In addition, as can be seen through the moderation modelling in the main text, congruency continues to be a significant covariate through all hypothesised and tested moderators. Together, under the GRADE guidelines, these factors indicate our confidence in the evidence can be increased.

# Supplement 8: Rating Models

## Table S8a

*Studies Included in Rating Models*

| Author(s) and year | Observers | Effects (*n*) |
| --- | --- | --- |
| Abramson et al. (2017) | 153 | 24 |
| Aviezer, Trope, and Todorov (2012a) | 45 | 20 |
| Israelashvili et al. (2019) | 245 | 3 |
| Ito et al. (2013) | 290 | 16 |
| Ko et al. (2011) | 110 | 24 |
| Namba et al. (2020) | 109 | 2 |
| Pollux et al. (2019) | 65 | 10 |
| Van Der Zant and Nelson (2021) | 132 | 16 |
| Xiao et al. (2016) | 32 | 8 |

## Figure S8a

*
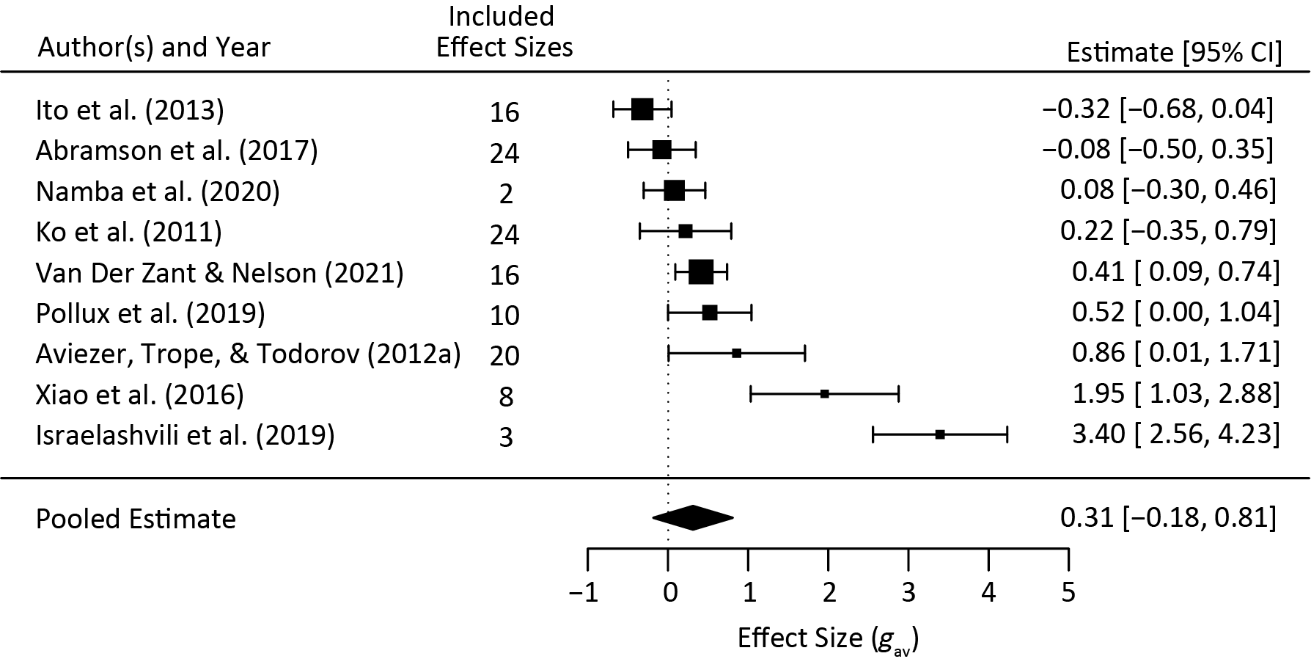
Forest Plot for Extracted Rating Effects*

## Table S8b

*Rating Baseline Model Fit*

| **Model** | ***g_av_* [95% CI]** | **SE** | ***t*** | ***p*** | **I^2^_(2)_ / I^2^_(3)_  (Q)** | **LRT** |
| --- | --- | --- | --- | --- | --- | --- |
| Initial Baseline Model |  |  |  |  |  |  |
| Two-level (*n* = 123) | 0.40 [-0.10, 0.89] | 0.20 | 1.98 | .097 | -/- (3919) | - |
| Three-level (*k* = 9, *n* = 123) | 0.54 [-0.07, 1.14] | 0.25 | 2.10 | .074 | .10/.89 (3919) | χ^2^ (1) = 1.77  *p* = .184 |

## Table S8c

*Rating Moderator Models*

| **Model** | ***vs reference levels*** | | | | **Wald omnibus** | |
| --- | --- | --- | --- | --- | --- | --- |
|  | ***g_av_* [95% CI]** | **SE** | ***t*** | ***p*** | ***F*** | ***p*** |
| **Reduced Model** (*k* = 9, *n* = 123)  I^2^_(2)_/ I^2^_(3)_ = 0.24/0.75; Q = 1,710; LRT = χ^2^(3) = 15.67, *p* = .001 | | | | |  |  |
| Intercept | 1.33 [0.06, 2.61] | 0.54 | 2.46 | .043 |  |  |
| **Effect of Faces (vs ref = Effect of context)** | **-1.37 [-4.33, 1.59]** | **0.95** | **-1.45** | **.241** | **9.44** | **.046** |
| Incongruent (vs ref = congruent effects) | -0.94 [-2.60, 0.71] | 0.49 | -1.93 | .159 | 5.45 | .096 |
| Congruency*Effect Type | -0.59 [-2.82, 1.64] | 0.88 | -0.68 | .528 |  |  |

*Note.* Reference levels for the intercept are congruent (congruency), and effect of context (effect type).

The *reduced model* found significant effects for effect type (*F*(2, 3.18) = 9.44, *p* = .046), but not congruence (*F*(2, 3.14) = 5.45, *p* = .095), whereby adding faces (*g_av_* = -.51) had a smaller effect on context, than context had on faces (*g_av_* = 1.04). However, neither the effect of faces nor the effect of context, were significantly larger than zero (*p*s > .164). The increased ratings when context is added indicates emotional context signals a higher intensity of emotion experience, while faces then tapper this intensity. This conceptualisation is made tentatively, requiring further investigation. Congruent and incongruent effects were numerically in the expected direction.

# Supplement 9: Reaction Time Models

## Table S9a

*Studies Included in Reaction Time Models*

| Author(s) and year | Observers | Effects (*n*) |
| --- | --- | --- |
| Aviezer, Hassin, & Bentin (2012) | 7 | 9 |
| Fenske and Eastwood (2003) | 40 | 6 |
| Hümmer et al. (2021) | 30 | 6 |
| Lecker and Aviezer (2021) | 277 | 18 |
| Li (2021) | 28 | 4 |
| Malaia et al. (2019) | 14 | 4 |
| Meeren et al. (2005) | 12 | 4 |
| Sasson et al. (2016) | 39 | 90 |
| Xu et al. (2017) | 15 | 4 |

## Figure S9a


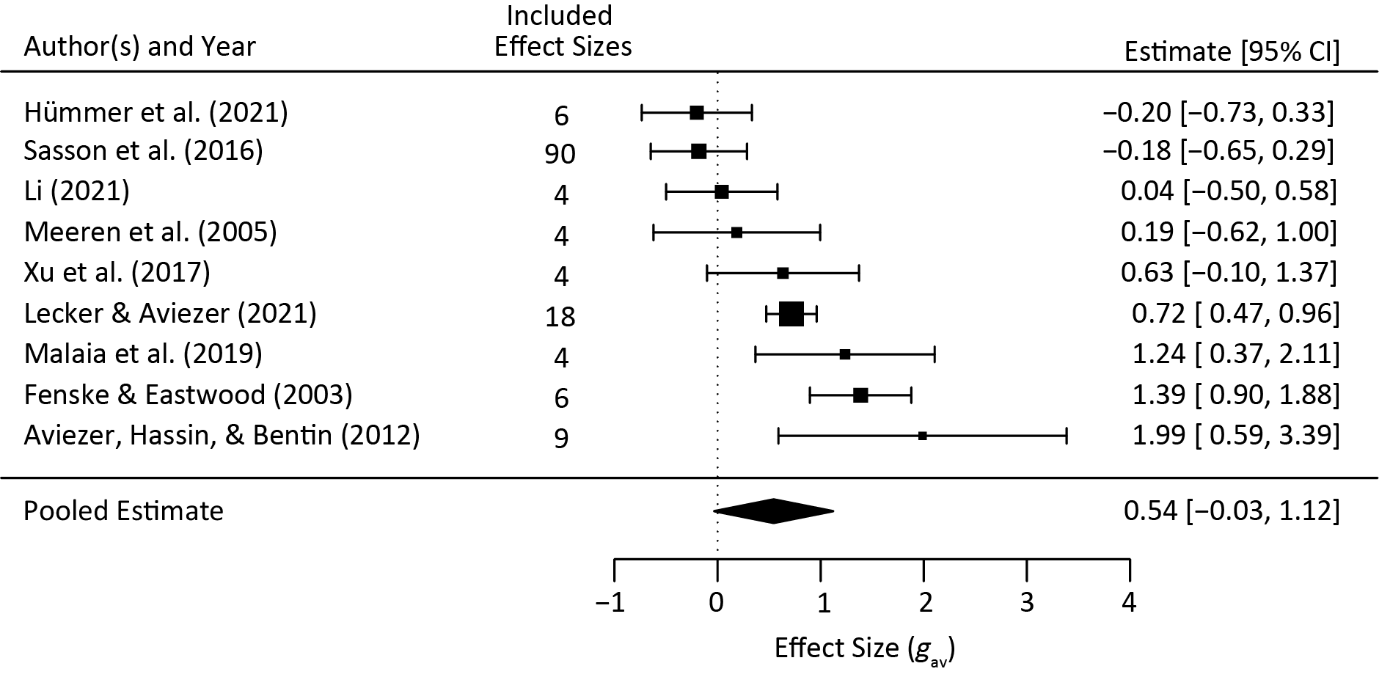
*Forest Plot for Reaction Time Effects*

## Table S9b

*Reaction Time Baseline Model Fit*

| **Model** | ***g_av_* [95% CI]** | **SE** | ***t*** | ***p*** | **I^2^_(2)_/ I^2^_(3)_**  **(Q)** | **LRT** |
| --- | --- | --- | --- | --- | --- | --- |
| *Initial Baseline Model* |  |  |  |  |  |  |
| Two-level (*n* = 145) | 0.16 [-1.45, 1.77] | 0.38 | 0.43 | .711 | -/- (2087) | - |
| Three-level (*k* = 9, *n* = 145) | 0.59 [0.03, 1.15] | 0.24 | 2.43 | .042 | .37/.59  (2087) | χ^2^ (1) = 36.20  *p* < .001 |

## Table S9c

*Reaction Time Moderator Models*

| **Model** | ***vs reference levels*** | | | | **Wald omnibus** | |
| --- | --- | --- | --- | --- | --- | --- |
|  | ***g_av_* [95% CI]** | **SE** | ***t*** | ***p*** | ***F*** | ***p*** |
| **Reduced Model** (*k* = 9, *n* = 145)  I^2^_(2)_/ I^2^_(3)_ = 0.33/0.63; Q = 1,710; LRT = χ^2^(3) = 18.99, *p* < .001 | | | | |  |  |
| Intercept | 0.29 [-0.14, 0.72] | 0.18 | 1.59 | .154 |  |  |
| Effect of Faces (vs ref = Effect of context) | -0.22 [-1.18, 0.75] | 0.19 | -1.15 | .386 | 1.44 | .648 |
| Incongruent (vs ref = congruent effects) | 0.64 [0.07, 1.21] | 0.19 | 3.36 | .037 | 12.25 | .187 |
| Congruency*Effect Type | -0.21 [-1.34, 0.92] | 0.24 | -0.86 | .488 |  |  |

*Note.* Reference levels for the intercept are effect type = effect of context, and congruency = congruent effects. LRT = Likelihood Ratio Test. Wald omnibus = estimated main effect of factor using supplemental omnibus Wald-type test.

The *reduced model* did not find significant effects for the effect of context (*g_av_* = 0.74; *F*(2, 4.11) = 5.07, *p* = .078), the effect of faces (*g_av_* = 0.38; *F*(2, 1.06) = 6.88, *p* = .247), congruent effects (*g_av_* = 0.22; *F*(2, 1.05) = 12.25, *p* = .187), or incongruent effects (*g_av_* = 0.79; *F*(2, 2.16) = 1.01, *p* = .490). Numerically, however, all four effects indicated slower reaction times for paired stimuli compared to isolated stimuli. These findings suggest integrative emotion perception requires greater cognitive processing than perceptual tasks of individual stimuli. This conceptualisation is made tentatively, requiring further investigation.

# Supplement 10: Incongruent Effects Across Emotion Pairings

During the review process, one reviewer raised the important question of whether the larger effect size for incongruent than congruent stimulus pairings was driven by certain emotion pairings having an outsized influence. To explore this possibility, we ran five separate models (one each for anger, disgust, fear, happy, and sad targets) to identify whether any incongruent emotion pairings produced significantly larger effects than other pairings.

Each model only included effects from a single target emotion (anger, disgust, fear, happiness, and sadness) and from incongruent pairings. The model predictors were effect type (face or context), emotion pairing (e.g., anger-disgust), and their interaction. We dropped clarity-difference scores from these models as there was no significant effect of clarity-difference for incongruent effects. Due to the number of comparisons required within each model, we applied a Bonferroni correction for each model (α = .05/12 = .004). As these models included a smaller number of effect sizes, we were not able to appropriately apply robust variance estimation to them and so the findings presented below do not include it.

Within each model, we ran pairwise comparisons between the emotion pairings within each effect type to identify any pairings that were significantly larger than others. The significant pairwise comparisons are presented below in Table S10, along with forest plots of each model (Figures S10a-S10e). We then re-ran the main model with these emotion pairings removed. Crucially, the main effect of congruency remained significant, *F*(3, 4.11) = 13.88, *p* = .013, with incongruent effects (*g_av_* = 2.85) significantly larger than congruent effects (*g_av_* = 0.74).

Table S10

*Comparisons Revealing Significant Differences in Effect Sizes Between Emotion Pairings*

| **Larger Effect Emotion Pair** | ***g_av_* (SE)** | **vs Significantly Smaller Effect Emotion Pair** | ***g_av_* (SE)** | ***t*** | ***p*** |
| --- | --- | --- | --- | --- | --- |
| Angry Context-Happy Face | 8.16 (1.65) | Angry Context-Disgusted Face | 1.58 (0.90) | 3.50 | < .001 |
| Disgusted Context-Sad Face | 3.75 (0.86) | Disgusted Context-Anger Face | -0.88 (1.38) | 3.19 | .002 |
| Disgusted Context-Happy Face | 4.90 (1.60) | Disgusted Context-Anger Face | -0.88 (1.38) | 3.04 | .004 |
| Disgusted Face-Angry Context | 3.59 (0.59) | Disgusted Face-Sad Context | 1.48 (0.68) | 3.66 | < .001 |
| Disgusted Face-Angry Context | 3.59 (0.59) | Disgusted Face-Fear Context | 0.71 (0.63) | 5.80 | < .001 |
| Happy Context-Sad Face | 16.25 (3.09) | Happy Context-Angry Face | 3.74 (3.09) | 3.04 | .004 |
| Sad Context-Angry Face | 10.94 (2.31) | Sad Context-Disgusted Face | 3.33 (2.14) | 3.26 | .002 |

## Figure S10a

*
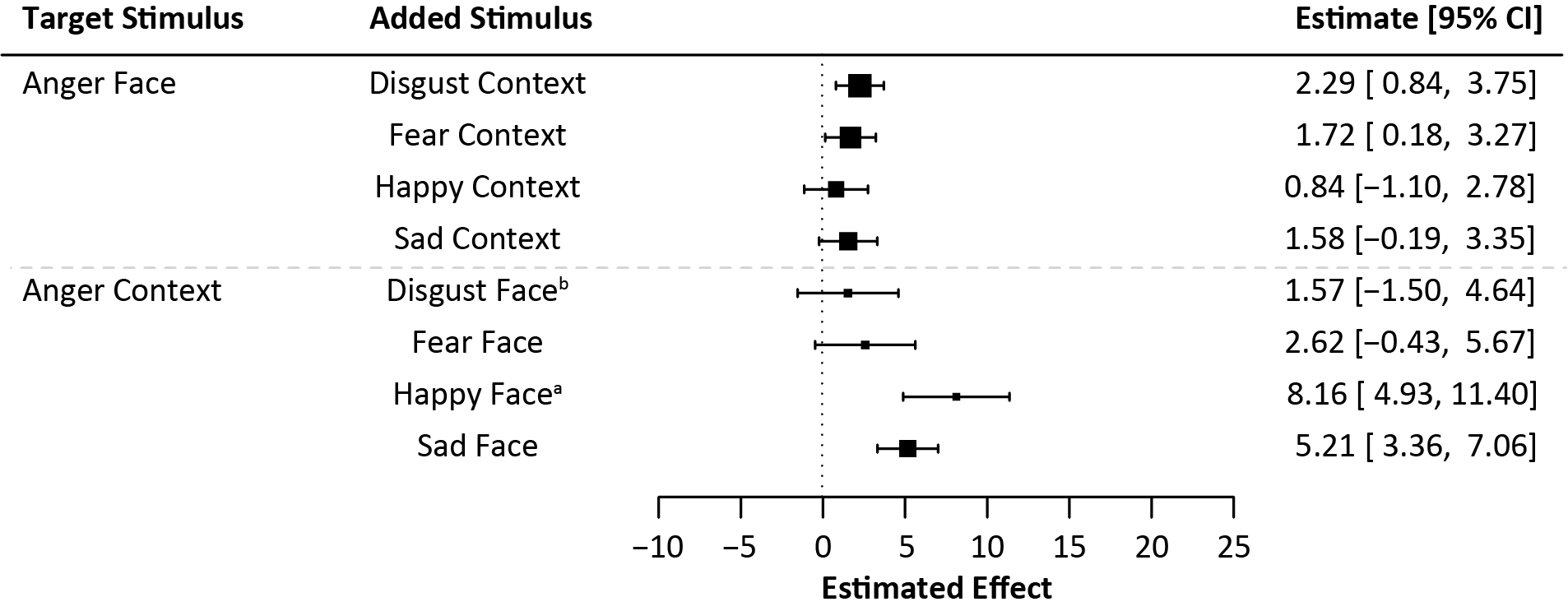
Forest Plot of Incongruent Emotion Pairs for Anger Targets*

*Note:* ^a^Indicates significantly larger effect for this pairing than for ^b^.

## Figure S10b

*Forest Plot of Incongruent Emotion Pairs for Disgust Targets*
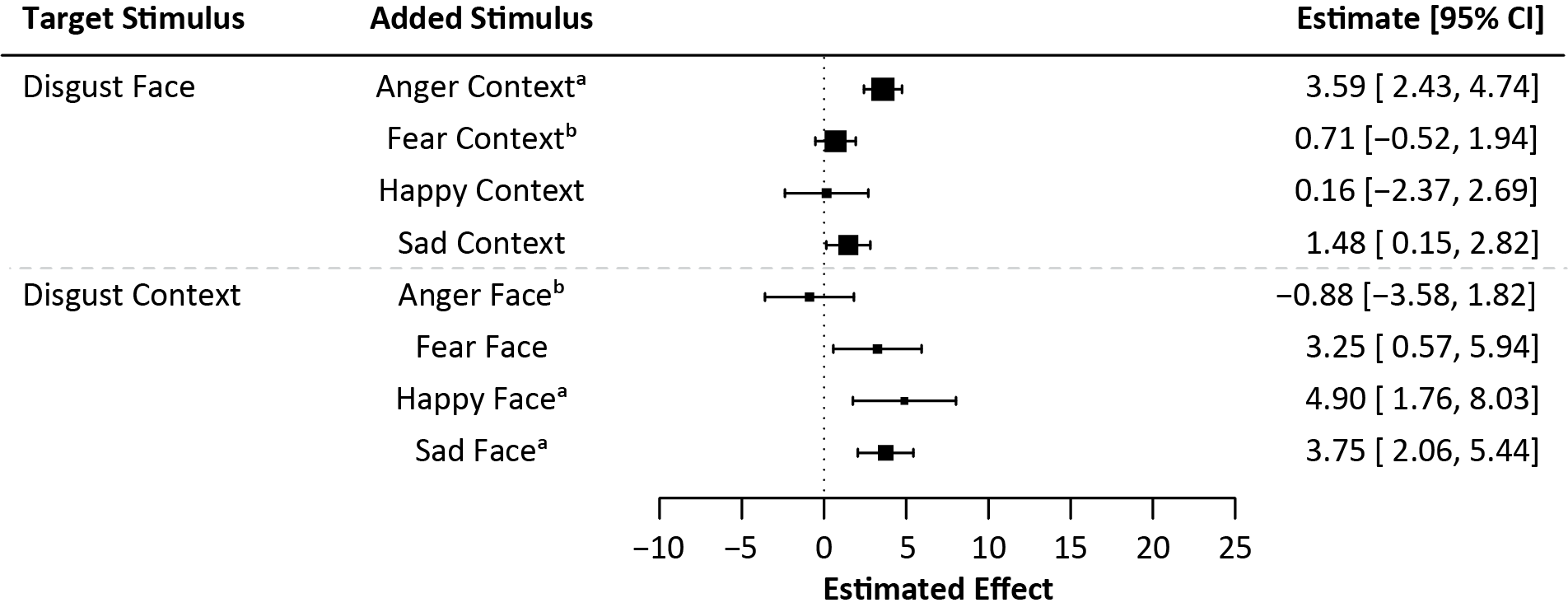


*Note:* ^a^Indicates significantly larger effect for this pairing than for ^b^.

## Figure S10c


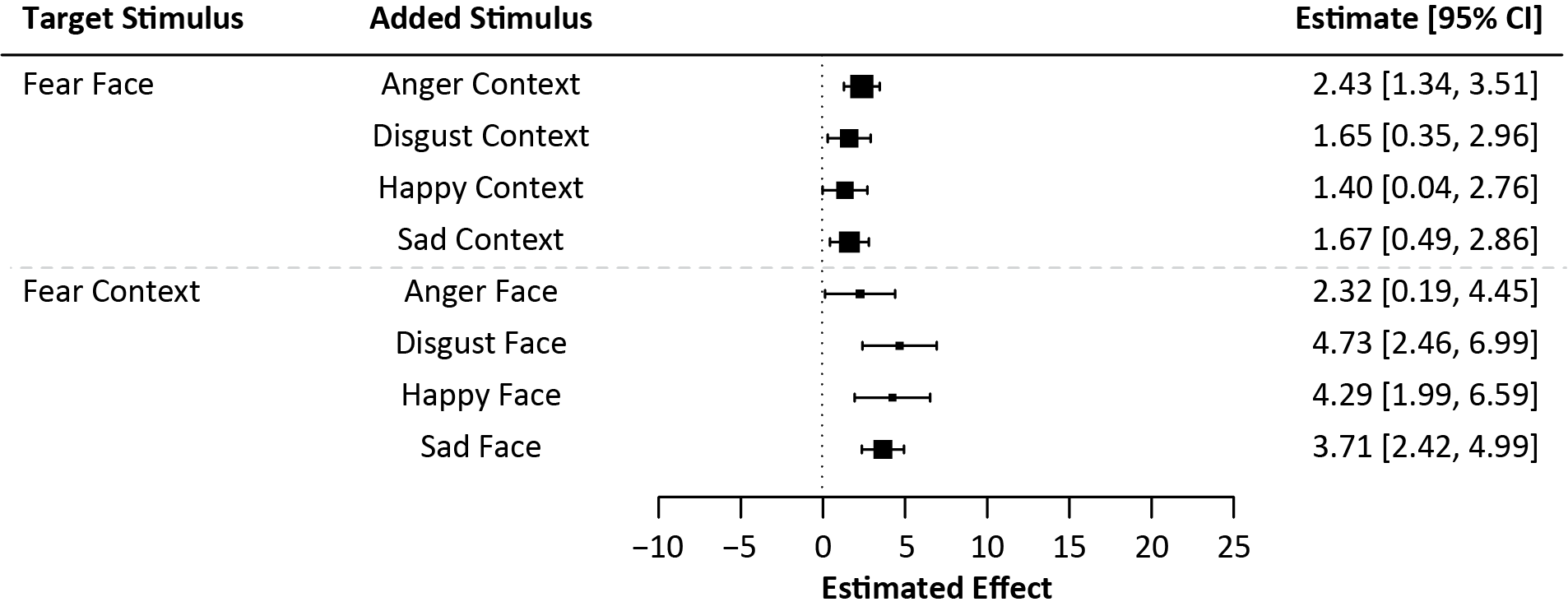
*Forest Plot of Incongruent Emotion Pairs for Fear Targets*

## Figure S10d


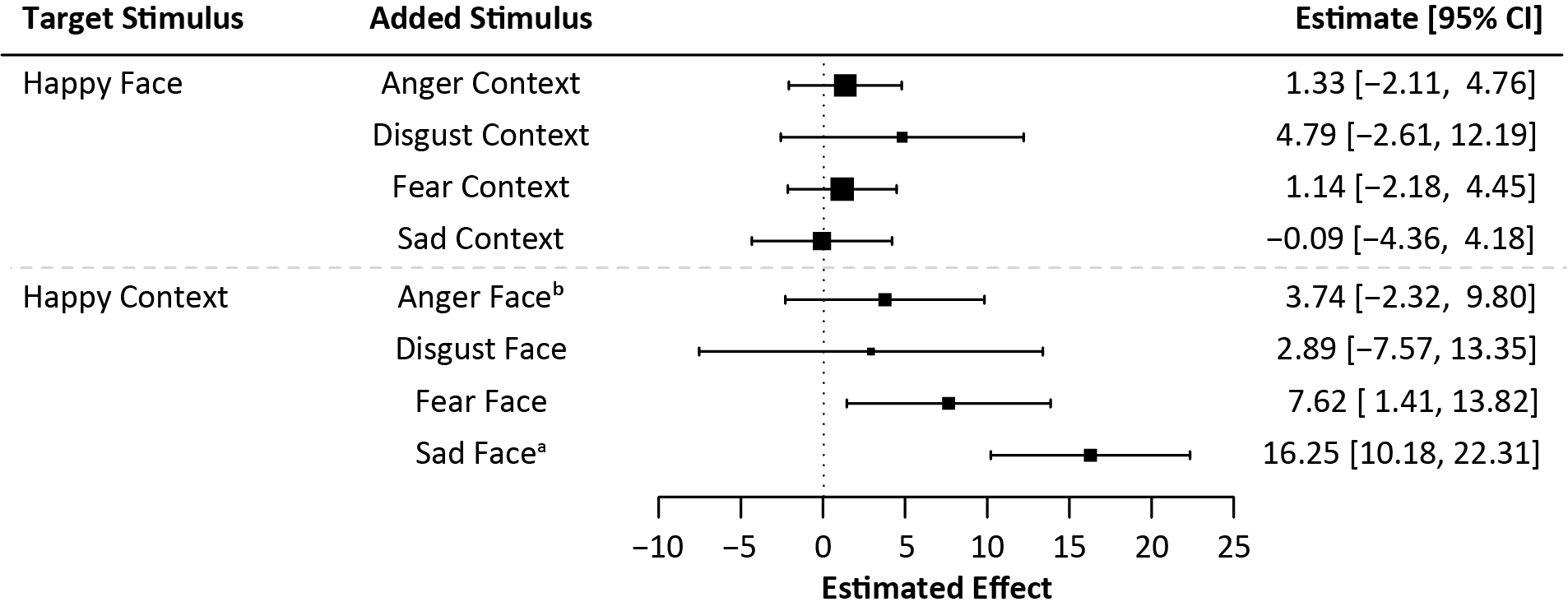
*Forest Plot of Incongruent Emotion Pairs for Happy Targets*

*Note:* ^a^Indicates significantly larger effect for this pairing than for ^b^.

## Figure S10e


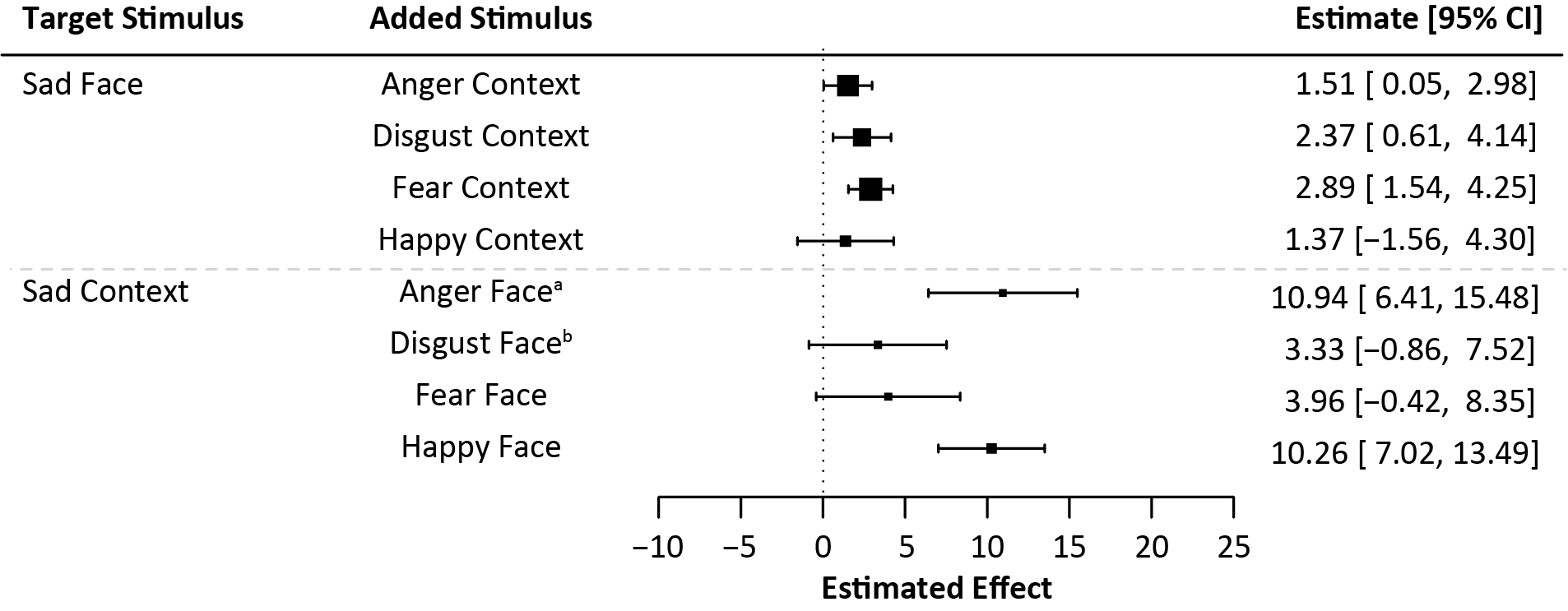
*Forest Plot of Incongruent Emotion Pairs for Sad Targets*

*Note:* ^a^Indicates significantly larger effect for this pairing than for ^b^.
